# Effects of binocularity and eye dominance on visually-driven ocular tracking

**DOI:** 10.3389/fnins.2025.1504628

**Published:** 2025-05-01

**Authors:** Kimia Seyedmadani, Keith A. Tucker, Mark R. Anderson, Yasemin M. Akay, Metin Akay, Leland S. Stone

**Affiliations:** ^1^Research Operations and Integration Group, Johnson Space Center, National Aeronautics and Space Administration, Houston, TX, United States; ^2^Department of Biomedical Engineering, University of Houston, Houston, TX, United States; ^3^Visuomotor Control Laboratory, Human Systems Integration Division, Ames Research Center, National Aeronautics and Space Administration, Moffett Field, CA, United States; ^4^Arctic Slope Regional Corporation, Federal Data Solutions, Moffett Field, CA, United States

**Keywords:** oculometric, smooth pursuit, saccades, binocular, monocular

## Abstract

**Introduction:**

We used 18 oculomotor performance metrics (oculometrics) to capture largely independent features of human ocular tracking. Our primary goal was to examine tracking eye movements in a healthy population under monocular and binocular viewing, as well as to examine the potential effects of line-of-sight eye dominance and spatial/directional tuning.

**Methods:**

We compared the ocular responses of 17 healthy well-rested participants using a radial step-ramp paradigm under three viewing conditions: both-eyes viewing, left-eye viewing, and right-eye viewing.

**Results:**

Our findings revealed that binocular viewing enhanced performance over that during monocular viewing for 11 oculometrics, with eye dominance associated with the selective enhancement of 3 oculometrics of visual motion processing. A comparison of binocular and dominant-eye viewing allowed us to segregate the direct enhancements of binocularity *per se* from those due simply to the inclusion of the dominant eye in binocular viewing and showed that viewing with two eyes is only directly responsible for the enhancement of 9 oculometrics. Our examination of spatial/directional tuning revealed largely isotropic enhancement due to binocularity, as well as several anisotropies in retinal functional processing: (1) a Nasal-Temporal asymmetry for pursuit latency and direction noise, and a Superior–Inferior asymmetry for latency, and (2) anisotropic enhancement in initial acceleration and direction noise (primarily for nasal retina) and speed noise (primarily for superior retina) when viewing through the dominant eye. We also documented Horizontal-Vertical anisotropies in initial acceleration, steady-state gain, proportion smooth, and speed responsiveness for both monocular and binocular viewing.

**Conclusion:**

Our findings demonstrate that there is isotropic enhancement from binocular viewing *per se* across a wide range of visuomotor features and that important normative characteristics of visual motion processing are shaped by retinal processing non-uniformly across visual space, modulated by eye dominance and perhaps related to previously found normative structural anisotropies in retinal thickness. This constellation of findings characterizes the subtle natural non-linear variations in visuomotor performance to provide insight into the relative roles of the retina and other brain areas in shaping visuomotor performance and to enable the detection of neurological and ophthalmological impairment through comparison with properly matched baselines in support of future research and clinical applications.

## Introduction

Visual and visuomotor function supports most human behavior, as the visual system dominates our perception of the environment around us and guides most of our voluntary and even reflexive actions. It has previously been shown that eye-movement behaviors can be used to capture variability across individuals in their visual and visuomotor performance capabilities (e.g., Hüttermann et al., 2018; Dalton, 2021). They also reveal differences within individuals across the normal healthy range due to everyday challenges (e.g., sleep or time of day, alcohol consumption, Stone et al., 2019; Tyson et al., 2021), as well as changes that may indicate neural injury (Liston et al., 2017; Mani et al., 2018; Stuart et al., 2020; McDonald et al., 2022) or pathology (Diefendorf and Dodge, 1908; Anderson and MacAskill, 2013; Leigh and Zee, 2015; Rempe et al., 2021; Alcañiz et al., 2022; Ahmed et al., 2022; Mylonas et al., 2022; Opwonya et al., 2022). The human visual system encompasses several pathways, all starting from the retina, (1) with most proceeding through the lateral geniculate of the thalamus and primary visual cortex culminating in extrastriate areas of the occipital, parietal, and frontal cortices (Mishkin et al., 1983; Merigan and Maunsell, 1993; Van Essen and Gallant, 1994; Wandell and Wade, 2003; Lennie and Movshon, 2005; Kandel et al., 2021), which contribute to visual motion perception (Albright, 1984; Britten et al., 1992; Krauzlis and Stone, 1999) and project to collicular, pontine, cerebellar, and brainstem areas to drive oculomotor responses (Krauzlis, 2004; Thier and Ilg, 2005; Lisberger, 2015; Robinson, 2022), (2) with other retinal projections going directly to brainstem structures (the Accessory Optic System) to drive optokinetic responses (Giolli et al., 2006) or to the superior colliculus then the pulvinar of the thalamus and ultimately to extra-striate cortical areas (Berman and Wurtz, 2008), and finally (3) with some emanating from both photoreceptors and intrinsically photosensitive ganglion cells of the retina to drive brainstem pupillary and circadian responses (Belliveau et al., 2024; Mure, 2021; Barrionuevo et al., 2023). Voluntary eye movements in particular have been proven to allow for the efficient collection of *oculometric* measures that are directly related to and, in some cases, indistinguishable from rigorous but time-consuming *psychophysical* measures of visual perception (Kowler and McKee, 1987; Beutter and Stone, 1998, 2000; Beutter et al., 2003; Watamaniuk and Heinen, 1999; Krauzlis and Stone, 1999; Eckstein et al., 2001; Stone and Krauzlis, 2003; Krukowski and Stone, 2005; Stone et al., 2000; Madelain and Krauzlis, 2003; Gegenfurtner, 2016). Saccade dynamics, the so-called main sequence (Bahill and Stark, 1979), provide insight into the functioning of the brainstem saccade generator and are known to be affected by mental fatigue (Scudder et al., 2002; Di Stasi et al., 2013; Chen et al., 2022). Similarly, the pupillary light reflex, provides insight into the functioning of non-image-forming subcortical visual pathways and is also known to be modulated by fatigue and arousal (Gamlin et al., 2007; Mure, 2021; Barrionuevo et al., 2023; Belliveau et al., 2024; Chen et al., 2022). Gaze holding ability during eccentric fixation provides insight into cerebellar and brainstem function (Zee et al., 1980; Leigh and Zee, 2015; Shemesh et al., 2021; Prsa and Thier, 2022). Thus, a broad set of ocular measures, including of pursuit, saccades, eccentric gaze holding, and pupillary light/dark responses, can be used to assess neural function across a wide range of visual pathways in the brain. Any divergence from the normal neural processing at any location along these pathways may manifest itself in some specific aspect of these ocular responses, with even small differences potentially indicating signs of subtle impairment. A solid baseline of normative performance is needed to enable across-subject testing when a within-subject baseline is not available.

Prior studies of nominal normative ocular tracking performance have largely examined binocular viewing conditions (e.g., Ke et al., 2013; Liston and Stone, 2014; Bargary et al., 2017; Stone et al., 2022), which provide useful baselines for the assessment of visual and oculomotor anomalies that reflect signals combined across both retinae starting with the primary visual cortex (Hubel and Wiesel, 1962). However, retinal pathologies, such as glaucoma, papilledema, macular degeneration, retinitis pigmentosa, and spaceflight neuro-ocular syndrome (Titchener et al., 2019; Lee et al., 2020; Crum et al., 2020; Verghese et al., 2021) typically impact the two eyes differently. Thus, while binocular performance baselines may be ideal for comparison with altered performance due to central stressors or pathology, there is a clear need for monocular performance baselines for comparison with altered function at the level of individual retinae for the detection of mild retinal impairment, with a potential to enhance the early diagnosis of retinal injury or pathology. The primary aim in this study is to characterize baseline ocular responses during monocular viewing using a previously established rapid oculometric test paradigm (Stone et al., 2019; Tyson et al., 2021) that efficiently harnesses a range of ocular subsystems (pursuit, saccades, gaze holding, pupillary light response) with the goal of enabling a future ability to detect subtle sub-clinical impairment of retinal function.

Prior binocular studies of ocular performance in the presence of mild stressors suggest that at least some monocular measures will likely be sensitive enough to reliably detect small visuomotor performance differences related to natural variation unrelated to pathology or injury. Thus, given that humans have dominant and non-dominant eyes (Miles, 1930; Fink, 1938) which, like handedness, may result in differences in performance when viewing monocularly with a dominant versus non-dominant eye (Ibi, 1997; Roth et al., 2002; Mapp et al., 2003; Cooper and Mendola, 2019; Ooi and He, 2020; Ramamurthy and Blaser, 2021; Kam and Chang, 2023), we examine here the most commonly described form of eye dominance, line-of-sight dominance (Miles, 1930; Mapp et al., 2003). As an extension of our primary goal of establishing oculometric performance baselines for monocular viewing, our second aim is to examine the effect of eye dominance because, to detect compromised retinal function, one may need to keep track of eye dominance as it is a likely contributor to natural visuomotor performance variation. Our third aim is to look for any response anisotropies across the retina as one may also need to keep track of retinal locus as this factor is also a likely contributor to performance variability. Lastly, our fourth aim is to compare performance under monocular viewing with that under binocular performance using the same established test paradigm (Stone et al., 2019; Tyson et al., 2021) to determine performance enhancements (if any) due to the engagement of a second eye *per se* (i.e., outside the context of stereovision) and to segregate any such effects from those due to eye dominance.

## Methods

### Ethical approval

The study was conducted at the National Aeronautics and Space Administration, Johnson Space Center, and was approved by the Human Research Institutional Review Board (NASA IRB) under protocol STUDY00000461. The investigation conformed to the latest revision of the Declaration of Helsinki, except for registration in a database, with each participant providing their informed, written consent prior to data collection.

### Selection/exclusion criteria

Our research subjects were obtained from the Johnson Space Center (JSC) clinic subject pool as healthy participants. Participants were adults aged 21–55 with corrected acuity of at least 20/40. They were prescreened in the JSC clinic for overall health, and in particular they were screened through Titmus^™^ testing (Cooper and Warshowsky, 1977) for color vision, depth perception, visual field, and interocular pressure anomalies. For twenty-four hours before running in the experiment, they had been asked to abstain from all caffeine, alcohol, and nicotine and, for the night preceding the test day, to have 8.5 h of lights-out, in-bed time for sleep. In addition, subjects self-reported to be free of neurological injury or illness, including but not limited to stroke, multiple sclerosis, Guillaume-Barre, epilepsy, myasthenia gravis, brain tumors, Traumatic Brain Injury (TBI), injury to the eye or orbit, diabetes, Attention-Deficit/Hyperactivity Disorder (ADHD), and insomnia. All participants were required to have regular sleep habits defined as Pittsburg Sleep Quality Index (PSQI) scores <5 and to be free of TBI with the Ohio State University TBI survey indicating no loss of consciousness or hospitalization due to head injury. The latter criterion eliminated one of the potential participants previously screened as healthy by JSC clinic.

### Procedures

Before their arrival, all participants received an introductory email reminding them of hours of sleep and the required abstinence from caffeine, nicotine, and alcohol before testing. In addition, participants were informed regarding the experiment timeline, which included 10 min to go over and sign the consent form, 5 min to fill out questionnaires, 10 min to perform the acuity and dominance eye-exams, 15 min to run the calibration and tracking task three times (monocular viewing with each eye alone then binocular) for a total of 70–80 min. We randomized the left and right monocular viewing order, but binocular viewing was always last.

Participants arrived at NASA JSC Research Operation and Integration laboratory (14 of our 17 participants between 7:30 and 8:00 a.m. with the remaining three arriving between 9:30 and 10:30 am). Upon arrival, they signed the approved informed consent form after being given the opportunity to read it and ask questions. After completing the health-history questionnaires (see above), a Snellen acuity test was performed, using a chart (line 8) viewed from a 20-foot distance was used to confirm a corrected visual acuity of 20/40 or better.

Participants were then asked to sit comfortably in an adjusted height chair with their chin in an ophthalmological chinrest to perform our visuomotor testing at a fixed viewing distance of 18.5″ (47 cm) with minimal head movement. The test apparatus consisted of a non-invasive 2D eye-tracker with high spatial precision (noise typically less than ¼ deg) using a high frame rate (250 Hz) camera (XIMEA™, Golden, CO) with a 75 mm macro lens (Computar™, Tokyo, Japan) positioned under the monitor. The light source was a 48-LED 850-nm illuminator situated next to the camera. Visual stimuli were presented on an HD-level resolution (1920×1080) monitor running at a refresh rate of 144 Hz (BenQ™ model XL2420Z). The chinrest could slide sideways to align the eye tracker for left or right eye viewing (we always recorded from the left eye during binocular viewing). During monocular viewing, participants used a disposable black eye patch to occlude the non-viewing eye for the 10 min needed to perform the tracking task monocularly.

After the participant was comfortably seated, the operator aligned the camera with the tracked eye and set the thresholding so that the camera could reliably track the pupil across the full range of gaze eccentricities tested. Participants then performed a calibration task using a 9-point cartesian grid (Beutter and Stone, 1998) with 3 additional fixations to test eccentric gaze holding and the pupillary light reflex (Tyson et al., 2021). Data were collected from the three tracking runs using an established testing paradigm (Stone et al., 2019). All our stimuli are restricted to the central ~10 deg within a single fronto-parallel plane at a fixed distance, so any binocular advantages are nearly exclusively due to the existence of a second complementary biological visual sensor (retina) for enhanced cyclopean vision, with no significant stereo depth cues from disparity or relative motion in the two eyes.

After the eye-movement testing was complete, participants performed a Miles test (Miles, 1930) to determine line-of-sight eye dominance. When viewing a partially occluded object binocularly, such that only one eye can view the target at a time, the eye that one naturally (unconsciously) points at the object (to avoid the occlusion), is the line-of-sight dominant eye. The Miles test was performed by having participants stand 20 feet away from an object on the wall, making a diamond-shaped aperture with their two hands at arm’s length, and looking at the object with both eyes open so that the object appears within the aperture. Participants were then asked to close their left eye and to report if the object remained visible or disappeared. If the object disappeared, participants were deemed left-eye dominant; if the object remained visible, they were deemed right-eye dominant. They were not told that this test was measuring eye dominance to avoid biasing their responses.

### Oculomotor task

**Figure 1:**
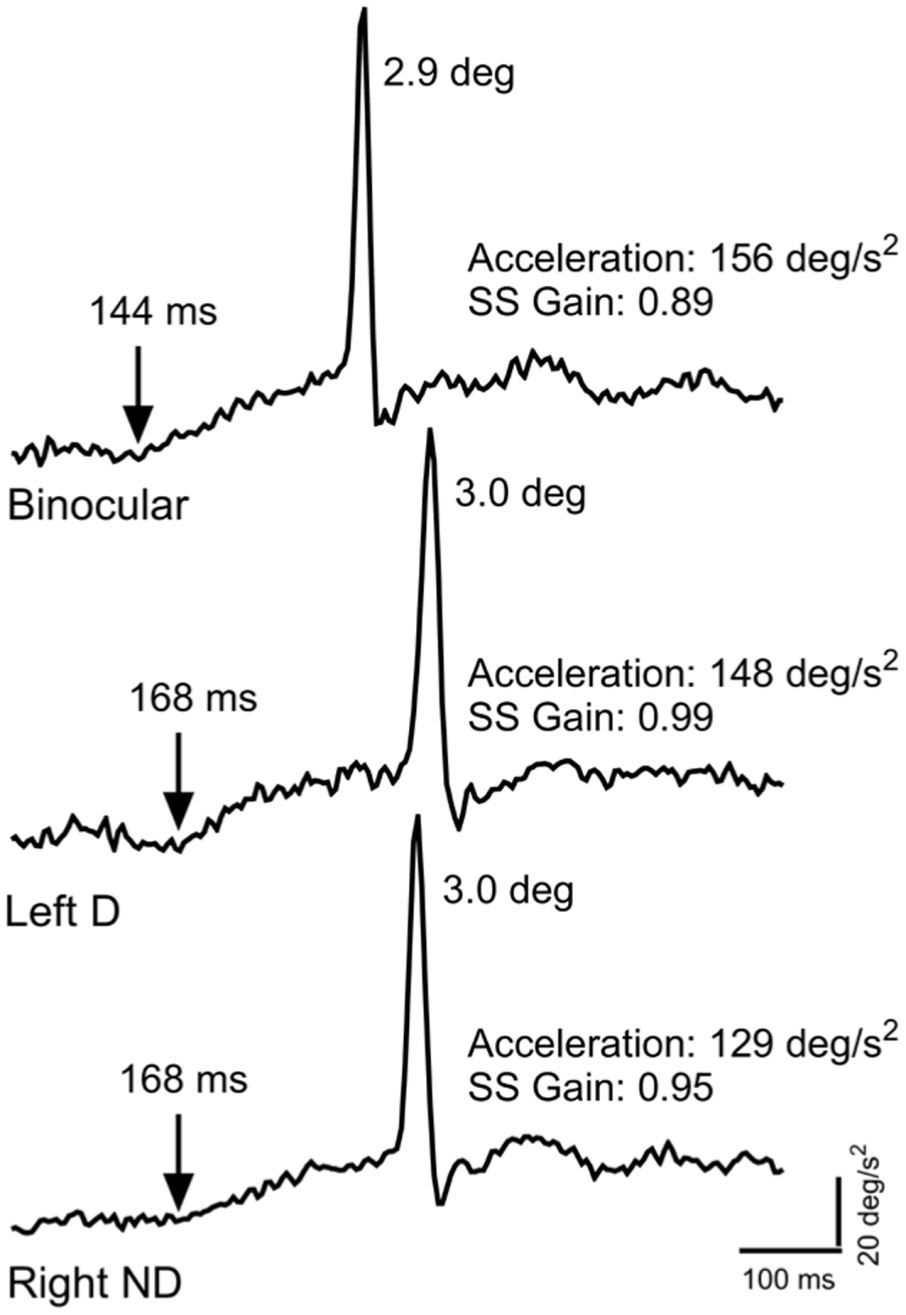
Typical raw eye-velocity traces from individual trials under three different viewing conditions for the same healthy participant. The top trace is the response to 22 deg/s ramp target motion 24 deg upward from pure leftward motion under binocular viewing. The middle trace is the response to 18 deg/s ramp target motion 24 deg downward from pure leftward motion under left (dominant) eye viewing. The bottom trace is the response to 20 deg/s ramp target motion 28 deg downward from pure rightward motion under right (non-dominant) eye viewing. Note that, while all three traces are qualitatively similar, they differ in important details. The binocular trace has a shorter pursuit latency compared to that in either monocular viewing condition. The two monocular conditions have similar pursuit latencies. Note also that the binocular and dominant eye responses have similarly brisk accelerations, while that for the non-dominant response is more sluggish. Lastly, the saccade amplitude (numbers adjacent to the saccadic velocity spike) for binocular viewing was slightly smaller compared to either monocular viewing condition. While the distributions of oculometric responses both within and across participants in these three conditions overlap considerably, the results in this figure illustrate the average behavior across our population. For examples of larger qualitative differences in oculometrics due to a behavioral stressor (see Figures 5A,B of Tyson et al., 2021).

The oculomotor paradigm used in this study has been described in detail previously (Stone et al., 2019; Tyson et al., 2021). Briefly, participants were asked to perform a radial Rashbass ocular tracking task (Rashbass, 1961; Krukowski and Stone, 2005; Liston and Stone, 2014). Each run consisted of 90 trials, with random directional sampling in 4° increments around the circle [0, 4, …, 356°]. Participants were instructed to fixate the central target and then to initiate the trial by a manual button press on a game controller when they were ready. They were encouraged to blink between trials. After a random amount of time between 200 and 5,000 ms (truncated exponential distribution), the target would jump in a random direction 3.2 to 4.8 deg away from the fixation point, immediately move back at a constant speed (randomly 16, 18, 20, 22, or 24 deg/s) toward the fovea, and then onwards for a random amount of time ranging from 700 to 1,000 ms before disappearing. Participants were instructed to keep their eyes on the stationary target in the center without blinking and then to follow it as best they could with their eyes once it started moving until it disappeared. The target would then reappear in the central location, awaiting the initiation of the next trial by the participant. We used the data from this paradigm, along with the calibration data, to generate 18 largely independent parameters of ocular (pupillary light reflex) and eye-movement (pursuit, saccades, visual motion processing, eccentric gaze holding) responses, in order to characterize the status of a number of brainstem, cerebellar, and cortical visual and visuomotor pathways (see Stone et al., 2019 for a discussion of the neurophysiological substrates of the various metrics). Raw data traces from three such ocular tracking trials are shown in Figure 1.

### Oculometric analyses

The 18 oculometrics parameters have been described in detail elsewhere and have been previously shown to be largely independent (Stone et al., 2019). They fall into 8 categories and are described briefly below. We computed them using in-house code written in MATLAB™ (R2020a, The MathWorks™, Natick, MA, USA).

- Early Perifoveal Vision:
  - Latency (ms) is defined as the median time across trials between stimulus motion onset and pursuit initiation (e.g., Tychsen and Lisberger, 1986a; Stone et al., 2019).
  - Initial Acceleration (deg/s^2^) is defined as the median eye acceleration in the first 100 ms immediately following initiation of pursuit (the “open-loop” period, Tychsen and Lisberger, 1986a).
- Late Foveal Vision:
  - Steady-State Gain is defined as the mean smooth radial eye speed projected onto the stimulus direction divided the velocity of the stimuli in the steady-state interval, i.e., 400–700 ms after motion onset (e.g., Robinson et al., 1986; Stone et al., 2019).
- Visual Motion Precision:
  - Direction Noise (°) is the local standard deviation of pursuit direction averaged across directions in the first 160-ms of pursuit (Krukowski and Stone, 2005).
  - Speed Noise (%) is the Weber fraction for speed estimation (Stone et al., 2019), analogous to that for perception (Kowler and McKee, 1987), i.e., the standard deviation of pursuit speed across trials at a given target speed divided by that target speed averaged across our set of target speeds in the steady-state interval.
- Visual Motion Accuracy:
  - Direction Anisotropy captures the four-fold “oblique-effect” non-linear distortion of direction estimation with direction differences around the cardinal directions exaggerated while those around the primary oblique directions diminished (Krukowski and Stone, 2005).
  - Direction Asymmetry captures the two-fold idiosyncratic “horizontal-vertical” non-linear distortion of direction discrimination with horizontal or vertical biases (Stone et al., 2019).
  - Speed Responsiveness is the best-fitting linear slope of the radial eye speed as a function of target speed (Stone et al., 2019).
- Subcortical Vision:
  - Saccade Amplitude (deg) is the mean angular distance between the beginning and end of forward catch-up saccades within the steady-state interval (Stone et al., 2019).
  - Saccade Rate (Hz) is the mean rate of catch-up saccade generation in the steady-state computed by dividing the total number of saccades by the total steady-state time (Stone et al., 2019).
  - Saccadic Dispersion (°) is the standard deviation of forward catch-up saccade direction across the steady-state interval (Stone et al., 2019).
  - Proportion Smooth is the mean proportion of time across trials that the steady-state interval is saccade-free (Stone et al., 2019).
- Pupillary Light Response:
  - Contraction *τ* (ms) is the primary time constant of the pupillary response to light onset (Tyson et al., 2017, 2021).
  - Dilation τ (ms) is the primary time constant of the pupillary response to light offset (Tyson et al., 2017, 2021).
  - Pupil Diameter (mm) is the mean pupil diameter across the entire on and off light cycle (Tyson et al., 2021).
- Brain Stem Saccade Generator:
  - Main Sequence Slope (Hz) is the best fitting linear-regression slope (Stone et al., 2019) of peak saccadic velocity as a function of saccade displacement curve (the so-called main sequence curve originally described by Bahill et al., 1975), corrected for the underlying pursuit response (De Brouwer et al., 2002).
  - Main Sequence Intercept (deg/s) is the best-fitting linear-regression intercept of the main sequence curve (Stone et al., 2019).
- Static Spatial Localization:
  - Fixation Error (deg) is the mean unsigned angular deviation between the fixation location and target location across the 9 points of the calibration grid.

### Data analysis and statistics

Using a within-subject design, we assessed participant performance as captured by our metrics by comparing three pairs of viewing conditions: (1) performance during monocular “cyclopean” viewing (the average of left-eye-only and right-eye-only viewing) versus binocular viewing, (2) performance during dominant-eye versus non-dominant-eye monocular viewing, and finally, (3) performance during dominant-eye versus binocular viewing. Firstly, we performed paired t-tests across participants in Excel™ for all metrics, except for the two pupillary response time constants where missing data (some participants blinked during the data collection obscuring the measurement) necessitated using unpaired tests. The assumption of a Gaussian distribution across participants underlying our use of t-tests for assessing the significance of observed differences across condition was valid most of the time (Shapiro–Wilk normality test, *p* > 0.05), and we labeled with an asterisk in the Tables any tentative *p*-values obtained for tests that failed the normality test. Secondly, for completeness and rigor, we also performed Wilcoxon Signed Rank and Wilcoxon Rank Sum (Mann–Whitney U tests) tests using GraphPad Prism™ to make non-parametric paired and unpaired, respectively, assessments of the significance of any observed differences between conditions. Thirdly, we performed simple conservative coin-flip tests using the tail of the binomial distribution to determine the probability of a given observation of at least N “heads” (defined as an observation of a condition effect with the expected sign without regard to the magnitude of the observed difference) given M “coin tosses” (defined as the total number of paired comparisons).

Throughout, we assumed the straightforward *a priori* hypothesis that binocular performance would be systematically superior to dominant monocular performance which would be superior to non-dominant monocular performance for all metrics (except for direction asymmetry and anisotropy, saccadic dispersion, and main sequence slope and intercept for which superior performance is ill-defined). This allowed us to use one-tailed testing if the sign of the observed effect was consistent with the *a priori* hypothesized sign. Furthermore, the effect of viewing condition differences on each metric was deemed a separate *a priori* independent hypothesis (i.e., is this particular metric the same or different across viewing conditions?) so we did not perform any corrections for the multiple metrics consistent with our prior studies (Stone et al., 2019; Tyson et al., 2021, 2023; Berneshawi et al., 2024). Lastly, although we randomly counterbalanced the testing order for the left and right eye viewing, we also controlled for any residual effect of test ordering by comparing the data when the dominant eye was tested first versus that when it was tested second using a two-tailed unpaired t-test across participants and found no significant difference (*p* > 0.05) for all 18 parameters.

### Analysis of the tuning curves across ramp-motion direction

We conducted a further spatial/directional analysis for seven of our metrics (Latency, Acceleration, Gain, Proportion Smooth, Direction Noise, Speed Noise, and Speed Responsiveness) at two levels of resolution, performing both quadrant and octant analyses, by subdividing each of our 90-trial sets of oculometric data into either 4 or 8 angular ranges. We were also interested in examining the data in retinal as well as world coordinates. We transformed the data from world into retinal coordinates by flipping the data from the left eye about the vertical axis to account for any potential systematic non-isotropic processing across the retina before combining data across the left and right eyes and thus to preserve any underlying Nasal-Temporal (N-T) asymmetry. We also tested for potential Horizontal-Vertical (H-V) and Superior–Inferior (S-I) asymmetries as well. Lastly, depending on whether the metric is associated with stimulus onset (associated with the retinal location generated by the step direction) or steady state (associated with the retinal location created by tracking in the target in the ramp direction), we would appropriately map from world visual field direction (L, R, U, D) to retinal coordinates (N, T, S, I).

For the quadrant analysis, we subdivided each of our 90-trial sets of oculometric data into quadrants. For example, all data collected for trials moving between 45 deg upward and downward of straight rightward were averaged and associated with the 0 deg (rightward) bin. We then performed paired two-tailed t-tests between pairs of quadrants (or averages across the two H or V quadrants) for each metric across subjects to determine if there was any N-T, S-I, or H-V asymmetry in retinal coordinates (for monocular viewing), or any R-L, or U-D, or H-V asymmetry in world coordinates (for binocular viewing). We Bonferroni-corrected the *p*-value for these three repeated t-tests used to look for any directional anisotropy without any specific prior hypothesis.

For the octant analysis, we subdivided each of our oculometric data into octants and plotted the resulting mean value (±SEM across subjects) in polar plots. For example, all data collected for trials moving between 22.5 deg upward and downward of straight rightward were averaged and associated with the 0 deg (rightward) octant. To determine conjointly the circularity of the left and right monocular polar plots, a χ^2^ test (df = 14, 8 directions x 2 eyes – 2) was performed by tallying the sum around the two circles of the mean squared deviation across subjects computed separately for each eye (thus allowing for random mean differences of the left and right eyes) normalized by its variance across subjects (the square of the standard error of the mean). To determine the circularity of the binocular polar plots, a χ^2^ test (df = 7, 8 directions – 1) was performed by tallying the sum around the circle of the mean squared deviation of the data normalized by its variance. To determine if there was a systematic difference between our viewing conditions, a χ^2^ test (df = 7, 8 directions – 1) comparison of the two conditions by tallying the sum around the circle of the mean squared differences between conditions normalized by its variance, which is just the sum of the variances of the two component means. The χ^2^ statistical computations were performed in Excel™ and the polar plots were generated with MATLAB™. In addition to the χ^2^ analysis above, we also performed paired two-tailed t-tests across subjects (df = 16) to determine if the polar plots for their left and right eyes were systematically shifted horizontally consistent with a significant N-T asymmetry.

## Results

### Participants

Our participant population (Table 1) was well-balanced by sex, ranged between 24 and 55 years of age, was neurologically healthy and well-rested (median/maximum Pittsburgh Sleep Index: 0.25/3.0), reported average of 7.7 h sleep, and voluntarily abstained from caffeine, alcohol, nicotine, and other recreational drugs prior to testing (see exclusion criteria in Methods). The ocular responses of this group can therefore be considered a normal human baseline.

**Table 1:** Participant characteristics.

	*N*	Age	Dominant eye	Best corrected acuity
Male	9	37 [24–53]	7L, 2R	<0.301 Logmar
Female	8	41.5 [28–55]	3L, 4R, 1 unknown	<0.301 Logmar

### Monocular vs. binocular viewing

Table 2 shows the parametric and non-parametric descriptive statistics for our 18 oculometric parameters, respectively, under binocular versus monocular viewing. As anticipated, many aspects of visuomotor function were significantly enhanced when viewing binocularly compared to when viewing monocularly. The parametric analysis indicates that participants have a significantly shorter mean latency (8.4%, *t*(16) = 8.135, *p* < 0.0001), higher mean initial acceleration (5.7%, *t*(16) = 2.132, *p* = 0.0244), lower mean saccade rate (13.9%, *t*(16) = 3.734, *p* = 0.0009), smaller mean saccade amplitude (16.3%, *t*(16) = 3.350 *p* = 0.0015), and higher mean proportion smooth (4.8%, *t*(16) = 2.861, *p* = 0.0038) for binocular viewing compared to monocular viewing. Participants also show faster mean pupillary contraction (9.8%, *t*(27) = 1.822, *p* = 0.0398) and dilation time constants (36%, *t*(30) = 1.908, *p* = 0.0330) with a smaller mean average pupil diameter (14%, *t*(16) = 3.591, *p* = 0.0012) for binocular viewing compared to monocular viewing. Finally, somewhat surprisingly, the saccadic main sequence slope was significantly lower with binocular viewing (11.7%, *t*(16) = 2.848, *p* = 0.0116). We also note that direction noise, anisotropy, and asymmetry all showed differences that approach significance. However, for the metrics with *p*-values labeled with an asterisk in Table 2, the normality test was not satisfied (i.e., Shapiro–Wilk test indicated *p* < 0.05), thus necessitating confirmation with non-parametric testing.

**Table 2:** Monocular vs. binocular analysis.

*N* = 17		Monocular Mean ± SD	Binocular Mean ± SD	*p* (t-test)	Monocular Median [range]	Binocular Median [range]	*p* (Wilcoxon)
Early Perifoveal Vision	Latency (ms)	165 ± 11	152 ± 11	**< 0.0001**	164 [144–182]	150 [132–176]	**< 0.0001**
	Acceleration (deg/s^2^)	117 ± 18	124 ± 16	0.0244*	116 [82–148.5]	124 [86–152]	**0.0149**
Late Foveal Vision	Gain	0.87 ± 0.08	0.86 ± 0.10	0.3405	0.90 [0.71–0.99]	0.86 [0.61–0.97]	0.3220
Visual Motion Precision	Direction Noise (◦)	9.5 ± 2.6	8.8 ± 3.2	0.0509	9.1 [5.6–14.3]	7.8 [5.0–15.5]	**0.0389**
	Speed Noise (%)	15.6 ± 2.7	15.3 ± 3.3	0.3233	15.4 [10.9–20.2]	15.1 [10.5–24.1]	0.2698
Visual Motion Accuracy	Direction Anisotropy	0.26 ± 0.11	0.35 ± 0.15	0.0676	0.24 [0.06–0.46]	0.35 [0.00–0.61]	0.106
	Direction Asymmetry	0.05 ± 0.13	0.12 ± 0.14	0.0692	0.05 (−0.22–0.32)	0.13 [−0.14–0.38]	**0.0485**
	Speed Responsiveness	0.53 ± 0.25	0.55 ± 0.22	0.2739	0.52 [0.18–0.93]	0.59 [0.12–0.88]	0.3777
Subcortical Vision	Saccade Rate (Hz)	3.49 ± 0.73	3.06 ± 0.83	**0.0010**	3.51 [2.26–4.72]	3.23 [1.63–4.38]	**0.0015**
	Saccade Amplitude (deg)	1.48 ± 0.48	1.27 ± 0.40	**0.0015**	1.50 [0.84–2.39]	1.15 [0.79–2.27]	**0.0013**
	Saccade Dispersion (◦)	16.8 ± 7.1	16.0 ± 7.5	0.4422	15.7 [10.1–41.4]	14.1 [8.3–38.8]	0.3683
	Proportion Smooth	0.78 ± 0.05	0.82 ± 0.08	0.0038*	0.76 [0.71–0.91]	0.79 [0.76–1.00]	**0.0020**
Pupillary Response	Contraction τ (ms)	178 ± 22	162 ± 25	0.0398*	176 [147–221]	156 [127–222]	**0.0314**
	Dilation τ (ms)	924 ± 439	677 ± 286	0.0330*	756 [485–2028]	588 [309–1,363]	**0.0135**
	Pupil Diameter (mm)	3.4 ± 0.4	2.9 ± 0.3	**0.0012**	3.3 [2.7–3.8]	2.9 [2.3–3.5]	**0.0010**
Brainstem Saccade Generator	Main Sequence Slope (Hz)	43.8 ± 8.9	39.2 ± 7.6	**0.0116**	45.0 [28.1–59.7]	40.7 [24.9–50.7]	**0.0194**
	Main Sequence Intercept (deg/s)	29.0 ± 10.2	31.9 ± 11.6	0.2866	29.9 [12.7–44.5]	32.2 [9.9–60.8]	0.3060
Localization	Fixation Error (deg)	0.57 ± 0.16	0.59 ± 0.40	0.7845	0.51 [0.36–0.89]	0.55 [0.18–1.88]	0.5394

This table shows the means and standard deviations as well as the medians and ranges across subjects of our 18 oculometric measures under monocular and binocular viewing along with parametric (*t*-test) and non-parametric (Wilcoxon test) comparisons. *p*-values with an asterisk indicate t-tests for which the normality criterion was not met. Bolded *p*-values indicate significant effects (*p* < 0.05).

The non-parametric analysis confirms that participants showed a significantly shorter median latency (8.4%, *W*(16) = −153, *p* = 0.0001), lower median initial acceleration (5.7%, *W*(16) = 91, *p* = 0.0149), lower median saccade rate (13.9%, *W*(16) = −119, *p* = 0.0015), smaller median saccade amplitude (16.3%, *W*(16) = 43, *p* = 0.0013), and higher median proportion smooth (4.8%, *W*(16) = 97, *p* = 0.0020) for binocular viewing than for monocular eye viewing. Again, participants showed faster median contraction (9.9%, *U*(28) = 61.5, *p* = 0.0314) and dilation time constants (36.5%, *U*(31) = 69, *p* = 0.0135) with a smaller median pupil diameter (15.0%, *W*(16) = −123, *p* = 0.0010), and lower median slope of the saccadic main sequence (4.9%, *W*(16) = −97, *p* = 0.0194) for binocular viewing than for monocular eye viewing. Lastly, two previously effects that were only marginally significant under parametric testing reached significance under non-parametric testing: the median direction noise (°) was lower (8.4%, *W*(16) = −75, *p* = 0.0389) and median direction asymmetry higher (58.3%, *W*(16) = 76, *p* = 0.0485) with binocular viewing.

**Figure 2:**
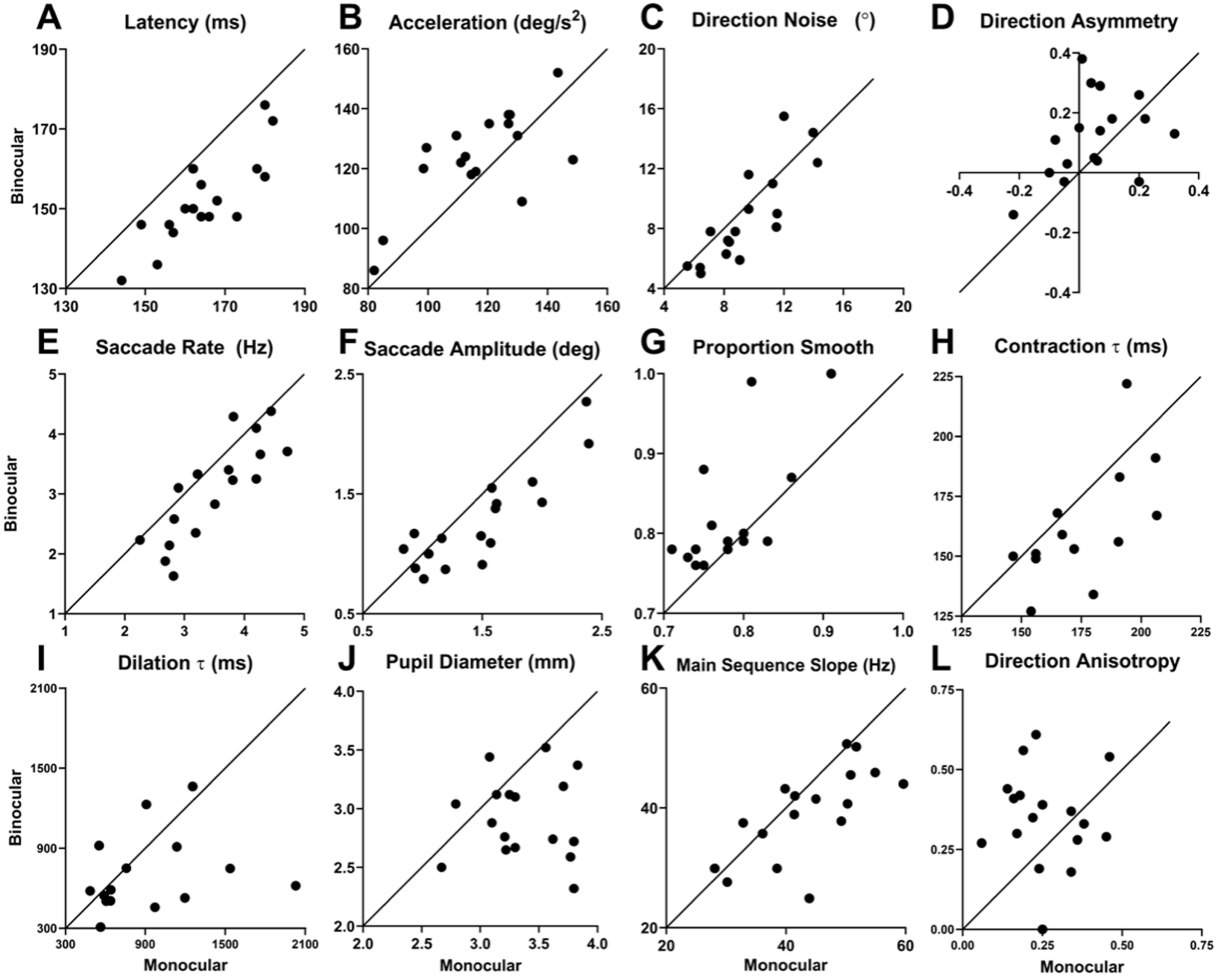
Plots of oculometric values under binocular vs. monocular viewing for **(A)** Latency, **(B)** Initial Acceleration, **(C)** Direction Noise, **(D)** Direction Asymmetry, **(E)** Saccade Rate, **(F)** Saccade Amplitude, **(G)** Proportion Smooth, **(H)** Contraction *τ*, **(I)** Dilation τ, **(J)** Pupil Diameter, **(K)** Main Sequence Slope, and **(L)** Direction Anisotropy. These plots provide visual evidence of the systematic ordinal differences between binocular vs. monocular viewing.

In summary, we found significant differences between performance under binocular and monocular viewing for 11 of the 18 oculometrics tested with the remaining metrics unchanged (Table 2). In plots of binocular values versus monocular values, the effects of binocular viewing become visually apparent (Figure 2). For latency, all 17 points are below the diagonal of slope 1 and intercept 0, indicating a highly significant effect using a simple coin-flip test and binomial statistics (*p* = 0.000008). For initial acceleration, there are 15 of 17 points on one side (*p* = 0.0012); for direction noise, 13 points (*p* = 0.0245); for direction asymmetry, 13 points (*p* = 0.0245); for saccade rate, 14 points (*p* = 0.0064); for saccade amplitude, 15 points (*p* = 0.0012); for proportion smooth, 13 points (*p* = 0.0245); for pupil diameter, 15 points (*p* = 0.0012), all indicating significant effects. However, the main-sequence slope with 12 points and direction anisotropy with 11 points both did not reach significance (*p* = 0.0717 and *p* = 0.1662, respectively). For the pupillary time constants, there were a few missing values which reduced the power of the coin-flip testing, however for the contraction *τ*, there were 10 out of 15 points on one side of the diagonal (*p* = 0.0592) and for the dilation τ, 10 out of 13 points (*p* = 0.0461), indicating nearly significant and significant effects, respectively. Thus, simple conservative coin-flip tests confirm the statistical findings between monocular and binocular viewing reported in Table 2, except for the main-sequence slope and the pupillary contraction time constant, for which coin-flip testing did not quite reach significance.

### Dominant vs. non-dominant viewing

Table 3 shows parametric and non-parametric descriptive statistics for our 18 oculometric parameters under viewing with the dominant and non-dominant eye. Only a few specific aspects of visuomotor function were superior when viewing with the dominant eye compared to when viewing with the non-dominant eye. In particular, mean initial acceleration is significantly faster (6.3%, *t*(15) = 1.981, *p* = 0.0331) and mean directional noise significantly lower (13.4%, *t*(15) = 3.089, *p* = 0.0037) with dominant-eye viewing. The non-parametric analysis confirms the significant increase in initial acceleration (6.3%, *W*(15) = −63, *p* = 0.0377) and decrease in direction noise (13.4%, *W*(15) = 107, *p* = 0.0018) with dominant-eye viewing. Lastly, the increase in speed responsiveness with dominant-eye viewing (14.4%, *W*(15) = 66, *p* = 0.0905) approaches significance.

**Table 3:** Dominant vs. non-dominant analysis.

*N* = 16		D Mean ± SD	ND Mean ± SD	*p* (t-test)	D Median [range]	ND Median [range]	*p* (Wilcoxon)
Early Perifoveal Vision	Latency (ms)	165 ± 12	163 ± 12	0.4329	167 [144–188]	160 [144–184]	0.4636
	Acceleration (deg/s^2^)	123 ± 20	115 ± 16	**0.0331**	127 [85–153]	120 [85–144]	**0.0377**
Late Foveal Vision	Gain	0.89 ± 0.08	0.87 ± 0.07	0.1436	0.90 [0.73–1.01]	0.88 [0.69–0.96]	0.1599
Visual Motion Precision	Direction Noise (◦)	8.6 ± 2.3	9.9 ± 2.8	0.0037*	8.6 [5.3–12.1]	9.7 (5.8–16.8)	**0.0018**
	Speed Noise (%)	15.2 ± 3.1	15.9 ± 3.2	0.1653*	15.3 [9.4–19.6]	15.7 [10.6–22.1]	0.1742
Visual Motion Accuracy	Direction Anisotropy	0.29 ± 0.13	0.23 ± 0.15	0.1618	0.29 [0.00–0.55]	0.23 [0.01–0.51]	0.1712
	Direction Asymmetry	0.04 ± 0.17	0.06 ± 0.17	0.713*	0.09 (−0.27–0.26)	0.03 [−0.17–0.38]	0.7339
	Speed Responsiveness	0.49 ± 0.28	0.58 ± 0.29	0.2035	0.43 [0.13–1.00]	0.59 [0.15–1.09]	0.1810
Subcortical Vision	Saccade Rate (Hz)	3.54 ± 0.74	3.41 ± 0.84	0.3051*	3.65 [2.27–4.85]	3.22 [2.24–4.76]	0.3484
	Saccade Amplitude (deg)	1.44 ± 0.56	1.46 ± 0.42	0.3143	1.43 [0.74–2.52]	1.48 [0.86–2.26]	0.3205
	Saccade Dispersion (◦)	16.6 ± 6.7	17.0 ± 8.2	0.6151	15.1 [11.4–39.3]	16.1 [8.7–43.4]	0.6407
	Proportion Smooth	0.77 ± 0.06	0.78 ± 0.06	0.5006	0.76 [0.67–0.90]	0.77 [0.71–0.92]	0.7723
Pupillary Response	Contraction τ (ms)	181 ± 34	173 ± 17	0.4788	175 [137–245]	175 [152–200]	0.3666
	Dilation τ (ms)	1,065 ± 743	780 ± 303	0.2160	721 [485–2,869]	627 [512–1,346]	0.6400
	Pupil Diameter (mm)	5.6 ± 0.6	5.8 ± 0.7	0.1727*	5.9 [4.4–6.7]	5.6 [4.8–6.8]	0.5124
Brainstem Saccade Generator	Main Sequence Slope (Hz)	42.7 ± 7.8	42.9 ± 9.6	0.9274*	42.2 [30.3–54.9]	42.3 [25.9–58.8]	0.8105
	Main Sequence Intercept (deg/s)	27.9 ± 10.1	29.1 ± 11.8	0.5232	29.3 [10.5–41.3]	29.6 [8.1–49.3]	0.3755
Localization	Fixation Error (deg)	0.57 ± 0.17	0.53 ± 0.2	0.5275*	0.53 [0.40–1.14]	0.52 [0.24–0.81]	0.5966

This table shows the means and standard deviations as well as the medians and ranges across subjects of our 18 oculometric measures under dominant and non-dominant eye viewing along with parametric (t-test) and non-parametric (Wilcoxon test) comparisons. *p*-values with an asterisk indicate t-tests for which the normality criterion was not met. Bolded *p*-values indicate significant effects (*p* < 0.05).

**Figure 3:**
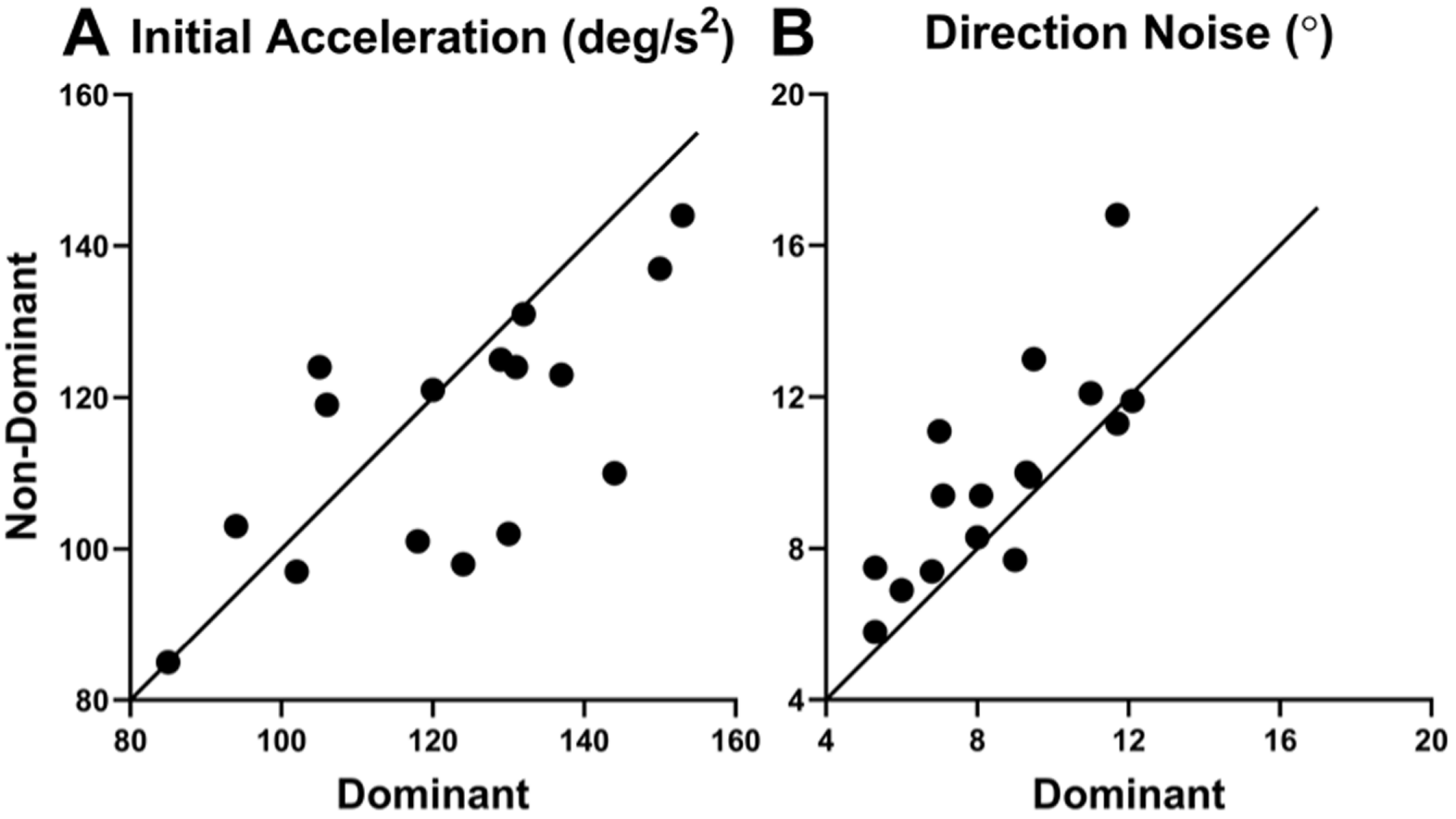
Plots of oculometric values under non-dominant vs. dominant viewing for **(A)** initial acceleration and **(B)** direction noise.

In summary, we found significant effects of dominant over non-dominant viewing for only 2 of our 18 oculometrics tested (Table 3). In plots of dominant values versus non-dominant values, again, the viewing effects are visually apparent (Figure 3). For initial acceleration, 11 of 16 points are below the diagonal of slope 1 and intercept 0, which does not quite reach significance using a simple coin-flip test (*p* = 0.1051). For direction noise, 13 of the 16 points are above the diagonal, indicating a significant effect (*p* = 0.0106). Thus, a simple conservative coin-flip test reaffirms the statistical finding of the significant difference between dominant and non-dominant eye viewing for direction noise, reported in Table 3, but only approaches significance for initial acceleration.

### Dominant vs. binocular viewing

To give monocular viewing its best chance to compete with binocular viewing, we also compared binocular viewing with monocular viewing from the dominant eye (as opposed the average of the two eyes as shown in Table 2). Table 4 shows parametric and non-parametric descriptive statistics for our 18 oculometric parameters under binocular and dominant-eye monocular viewing. The parametric analysis indicates a significantly shorter mean latency (9.6%, *t*(15) = 7.151, *p* < 0.0001), a significantly lower mean saccade rate (16.5%, *t*(15) = 3.381 *p* = 0.0021) and amplitude (15.0%, *t*(15) = 2.517, *p* = 0.0118), and a significantly higher mean proportion smooth (5.5%, *t*(15) = 2.935, *p* = 0.0051) for binocular viewing compared to dominant-eye viewing. Participants also showed faster mean pupillary contraction (13.2%, *t*(30) = 1.751, *p* = 0.0466) and dilation time constants (58.3%, *t*(30) = 3.251, *p* = 0.0014) with a smaller mean average pupil diameter (13.9%, *t*(15) = 5.142, *p* < 0.0001) with binocular viewing. Finally, the saccadic main sequence slope was significantly lower with binocular viewing (9.94%, *t*(15) = 2.196, *p* = 0.0443). We also note that direction asymmetry and speed responsiveness effects fell just below significance (*p* = 0.0553 and *p* = 0.0810, respectively).

**Table 4:** Dominant vs. binocular analysis.

*N* = 16		Dominant Mean ± SD	Binocular Mean ± SD	*p* (t-test)	Dominant Median [range]	Binocular Median [range]	*p* (Wilcoxon)
Early Perifoveal Vision	Latency (ms)	165 ± 12	150 ± 10	<0.0001*	167 [144–188]	149 [132–172]	**<0.0001**
	Acceleration (deg/s^2^)	123 ± 20	126 ± 13	0.1915	127 [85–153]	126 [96–152]	0.2799
Late Foveal Vision	Gain	0.89 ± 0.08	0.87 ± 0.08	0.3520	0.90 [0.73–1.01]	0.86 [0.71–0.97]	0.4484
Visual Motion Precision	Direction Noise (◦)	8.6 ± 2.3	8.4 ± 2.3	0.3678	8.6 [5.3–12.1]	7.8 [5.0–15.5]	0.365
	Speed Noise (%)	15.2 ± 3.1	15.3 ± 3.4	0.4225	15.3 [9.4–19.6]	14.7 [10.5–24.1]	0.445
Visual Motion Accuracy	Direction Anisotropy	0.29 ± 0.13	0.34 ± 0.15	0.3281	0.29 [0.00–0.55]	0.34 [0.00–0.61]	0.4257
	Direction Asymmetry	0.04 ± 0.17	0.11 ± 0.13	0.0553	0.09 (−0.27–0.26)	0.12 [−0.14–0.38]	0.0717
	Speed Responsiveness	0.49 ± 0.28	0.57 ± 0.22	0.0810	0.43 [0.13–1.00]	0.59 [0.12–0.88]	0.0776
Subcortical Vision	Saccade Rate (Hz)	3.54 ± 0.74	3.04 ± 0.86	**0.0041**	3.65 [2.27–4.85]	3.17 [1.63–4.38]	**0.0042**
	Saccade Amplitude (deg)	1.44 ± 0.56	1.25 ± 0.40	**0.0118**	1.43 [0.74–2.52]	1.14 [0.79–2.27]	**0.0162**
	Saccade Dispersion (◦)	16.6 ± 6.7	16.1 ± 7.8	0.5769	15.1 [11.4–39.3]	13.9 [8.3–38.8]	0.411
	Proportion Smooth	0.77 ± 0.06	0.82 ± 0.08	**0.0051**	0.76 [0.67–0.90]	0.79 [0.76–1.00]	**0.0061**
Pupillary Response	Contraction τ (ms)	181 ± 34	160 ± 25	0.0466*	175 [137–245]	155 [127–222]	0.0792
	Dilation τ (ms)	1,065 ± 743	673 ± 295	0.0325*	721 [485–2,869]	584 [309–1,363]	0.0715
	Pupil Diameter (mm)	3.3 ± 0.4	2.9 ± 0.3	**0.0001**	3.4 [2.6–3.9]	2.8 [2.3–3.5]	**<0.0001**
Brainstem Saccade Generator	Main Sequence Slope (Hz)	42.7 ± 7.8	38.9 ± 7.7	0.0443*	42.2 [30.3–54.9]	39.8 [24.9–50.7]	0.0522
	Main Sequence Intercept (deg/s)	27.9 ± 10.1	30.1 ± 9.2	0.3509	29.3 [10.5–41.3]	31.35 [9.9–43.6]	0.3825
Localization	Fixation Error (deg)	0.57 ± 0.17	0.51 ± 0.22	0.3822	0.53 [0.40–1.14]	0.47 [0.18–0.98]	0.3755

This table shows the means and standard deviations as well as the medians and ranges across subjects of our 18 oculometric measures under dominant and binocular viewing along with parametric (t-test) and non-parametric (Wilcoxon test) comparisons. *p*-values with an asterisk indicate t-tests for which the normality criterion was not met. Bolded *p*-values indicate significant effects (*p* < 0.05).

The non-parametric analysis leads to similar findings of a significantly shorter latency (9.6%, *W*(15) = −132, *p* < 0.0001), lower saccade rate (16.5%, *W*(15) = −90, *p* = 0.0042), smaller saccade amplitude (15.0%, *W*(15) = −82, *p* = 0.0118), and higher proportion smooth (5.5%, *W*(15) = 62, *p* = 0.0061) with binocular viewing compared to dominant-eye viewing. It also indicates a smaller pupil diameter (13.9%, *W*(15) = −130, *p* < 0.0001). The decreases in pupillary contraction and dilation time constants approached significance (*U*(24) = 45.5, *p* = 0.0792 and *U*(28) = 64, *p* = 0.0715, respectively) as did that in the slope of the main sequence (*W*(16) = −75, *p* = 0.0522). Thus, we found clear significant effects of binocular versus dominant-eye viewing for 5 of the 18 oculometrics tested with 4 more showing hints of effects.

**Figure 4:**
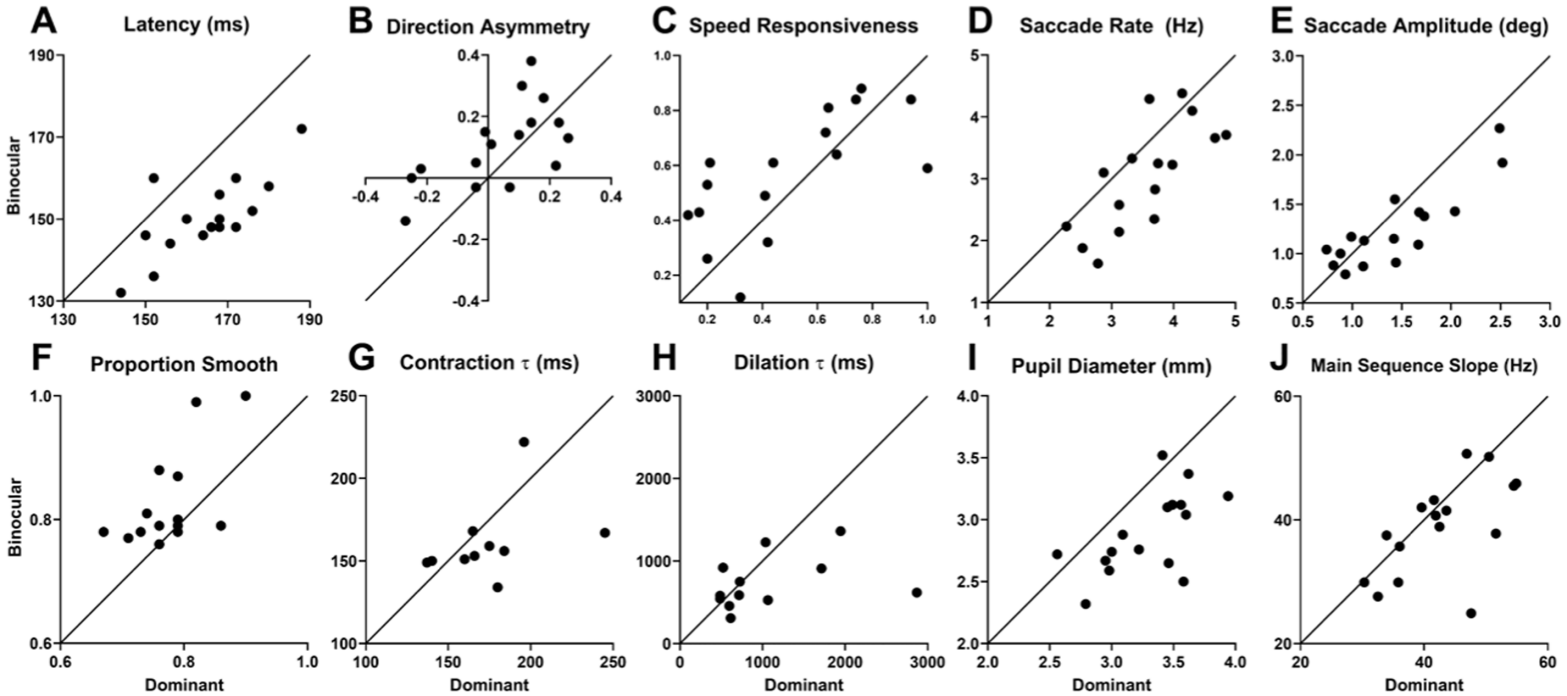
Plots of oculometric values under binocular vs. dominant eye viewing for latency **(A)**, direction asymmetry **(B)**, speed responsiveness **(C)**, saccade rate **(D)**, saccade amplitude **(E)**, proportion smooth **(F)**, pupillary contraction time constant **(G)**, dilation time constant **(H)**, mean pupil diameter **(I)**, and main-sequence slope **(J)**.

From plots of binocular values versus dominant values, the effects of binocular viewing appear more expansive (Figure 4). For latency, 15 of 16 points are on one side of the diagonal of slope 1 and intercept 0, indicating a significant effect using a simple coin-flip test (*p* = 0.0003); for saccade rate, 12 points (*p* = 0.0384); for proportion smooth, 14 points (*p* = 0.0021); for direction asymmetry, 12 points (*p* = 0.0384); for pupil diameter, 14 points (*p* = 0.0021); and for main sequence slope, 12 points (*p* = 0.0384), all indicating significance. Thus, simple conservative coin-flip tests largely reaffirm the statistical findings of the significant differences between dominant-eye and binocular viewing reported in Table 4, except that saccadic amplitude failed to reach significance (*p* = 0.2273) and two previously marginal effects of direction asymmetry and main-sequence slope became significant. Hampered by missing data, the statistical power, even with coin-flip testing, for the pupillary time contraction and dilation constants remained too weak to detect significant enhancement of binocular over dominant viewing. However, importantly, binocular viewing shows a clear significant enhancement over monocular viewing with no significant difference between dominant and non-dominant viewing (see above).

### Segregating the effects of line-of-sight eye dominance and binocularity

**Figure 5:**
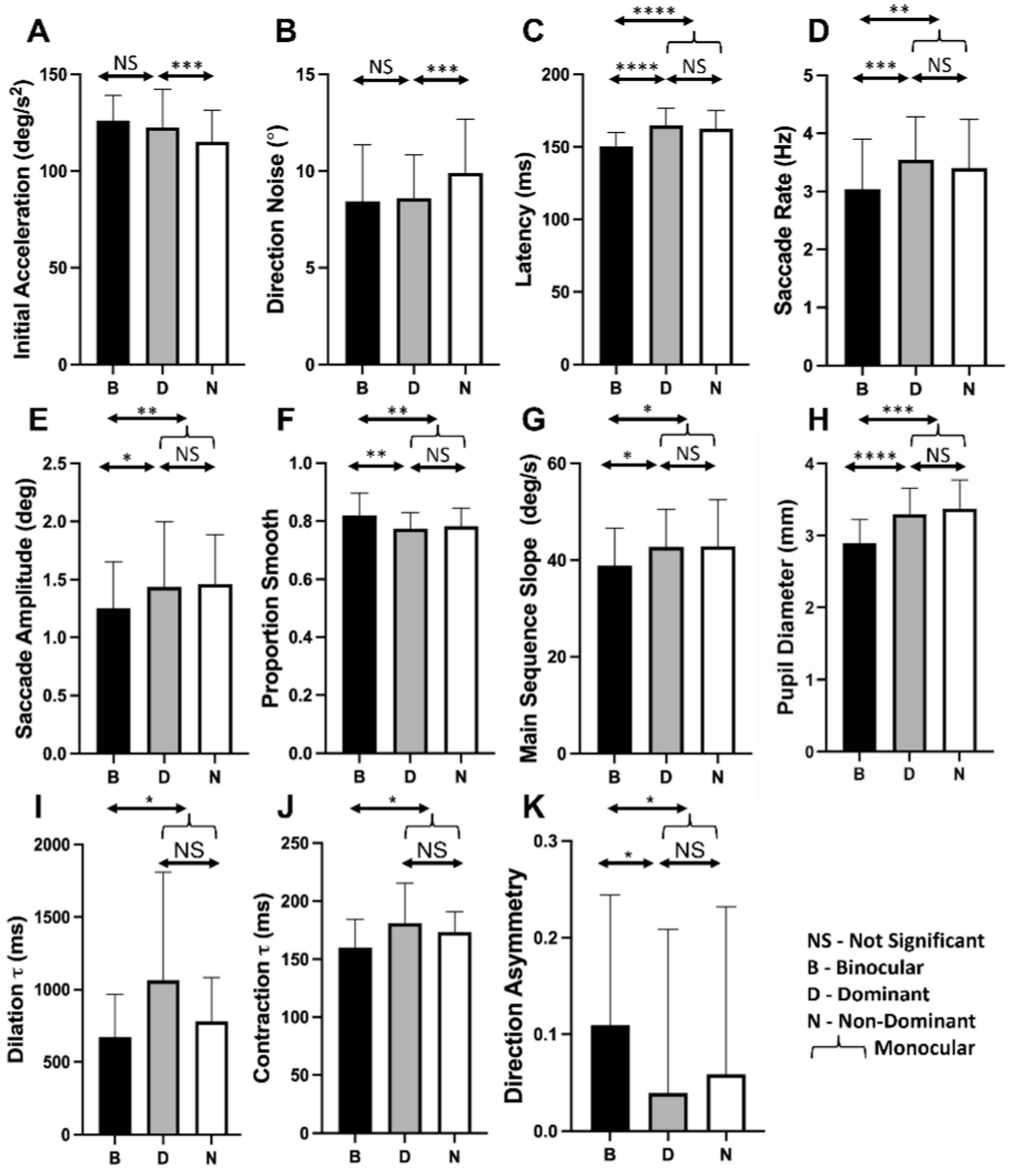
Histograms of oculometric values for binocular (B-black), dominant (D-gray), and non-dominant (N-white) eye viewing for **(A)** initial acceleration, **(B)** direction noise, **(C)** latency, **(D)**, saccade rate, **(E)** saccade amplitude, **(F)** proportion smooth, **(G)** main-sequence slope, **(H)** pupil diameter, **(I)** dilation τ, **(J)** contraction τ, and **(K)** direction asymmetry. Statistical measures are from the statistical analysis above with NS, *, **, ***, **** indicating >0.05, <0.05, <0.01, <0.005, and <0.001, respectively.

Figure 5 compares oculometric performance across our three main viewing conditions summarizing the statistical analyses above. Initial acceleration and direction precision are enhanced by dominant-eye viewing with no additional binocular advantage. Conversely, latency, saccade rate, saccade amplitude, proportion smooth, main-sequence slope, direction asymmetry, pupillary contraction and dilation time constants, mean pupil diameter are all altered by binocular viewing *per se*, independent of eye-dominance effects.

### Spatial/directional analysis of oculometrics of visual motion processing

#### Quadrant analysis

We performed a crude spatial/directional tuning analysis of our data for seven pursuit-related oculometrics by dividing the data into direction quadrants. Table 5 (top) shows the mean and standard deviation across all participants of latency, acceleration, gain, proportion smooth, direction noise, speed noise, and speed responsiveness associated with stimulation of the nasal, superior, temporal, and inferior perifoveal retina under monocular viewing. We defined retinal coordinates for measures of tracking initiation (latency, initial acceleration, direction noise) by the direction of the stimulus step on the retina (with the eye by design leading the target motion throughout the initiation analysis period). We defined retinal coordinates for measures of steady-state tracking by the direction of the stimulus ramp (because the eye typically lags the target at least slightly during the steady-state analysis period). For latency, we found significant N-T (*t*(16) = −2.357, *p* = 0.0315) and S-I (*t*(16) = 3.138, *p* = 0.0064) asymmetries, with systematically shorter latencies (i.e., better performance) for motion stimuli presented on nasal and inferior, as opposed to temporal and superior, portions of the parafoveal retina, respectively. Direction noise showed an N-T asymmetry that nearly reached significance (*t*(16) = −2.0504, *p* = 0.0571) with potentially more precise directional responses for motion stimuli on the nasal parafoveal retina. Initial acceleration (*t*(16) = 2.869, *p* = 0.0111), steady-state gain (*t*(16) = 10.929, *p* < 0.0001), proportion smooth (*t*(16) = 2.668, *p* = 0.0169), and speed responsiveness (*t*(16) = 5.533, *p* < 0.0001) all showed a significant H-V asymmetry, resulting in smoother responses with systematically more vigorous initial acceleration, more accurate steady-state gain and greater speed sensitivity for motion stimuli along or near the horizonal, as compared to the vertical, meridian. The H-V asymmetry of latency almost reached significance (*t*(16) = −2.003, *p* = 0.0625).

**Table 5:** Mean and standard deviation across our population of 17 participants of their monocular performance averaged across both eyes in retinal coordinates (top half) and of their binocular performance in world coordinates (bottom half).

	Latency (ms)	Initial acceleration (deg/s^2^)	Gain	Proportion smooth	Direction noise (⁰)	Speed noise (%)	Speed responsiveness
Monocular Retinal	Mean ± SD	Mean ± SD	Mean ± SD	Mean ± SD	Mean ± SD	Mean ± SD	Mean ± SD
Nasal (N)	160 ± 12	130 ± 24	0.92 ± 0.08	0.81 ± 0.07	10.4 ± 2.5	10.5 ± 3.6	0.58 ± 0.28
Superior (S)	173 ± 14	118 ± 19	0.80 ± 0.12	0.77 ± 0.06	11.0 ± 3.0	11.9 ± 2.6	0.44 ± 0.21
Temporal (T)	166 ± 13	127 ± 19	0.92 ± 0.10	0.79 ± 0.07	11.5 ± 2.8	11.1 ± 3.1	0.63 ± 0.21
Inferior (I)	163 ± 12	117 ± 19	0.73 ± 0.10	0.74 ± 0.09	10.9 ± 2.8	12.1 ± 2.8	0.34 ± 0.30
Binocular Directional	Mean ± SD	Mean ± SD	Mean ± SD	Mean ± SD	Mean ± SD	Mean ± SD	Mean ± SD
Right (R)	148 ± 13	133 ± 18	0.92 ± 0.12	0.83 ± 0.06	9.8 ± 3.7	9.6 ± 2.8	0.72 ± 0.29
Up (U)	159 ± 15	123 ± 22	0.72 ± 0.18	0.76 ± 0.10	9.8 ± 3.8	12.1 ± 3.4	0.36 ± 0.25
Left (L)	153 ± 13	142 ± 19	0.89 ± 0.11	0.83 ± 0.07	10.1 ± 4.0	11.1 ± 4.9	0.56 ± 0.24
Down (D)	149 ± 10	121 ± 27	0.81 ± 0.13	0.79 ± 0.07	10.5 ± 4.4	12.2 ± 3.3	0.34 ± 0.25

Table 5 (bottom) shows the mean and standard deviation across all participants of latency, acceleration, gain, proportion smooth, direction noise, speed noise, and speed responsiveness associated with rightward, leftward, upward, and downward motion in world/head coordinates under binocular viewing. For latency, we found significant U-D asymmetry (*t*(16) = 2.582, *p* = 0.0201) with systematically shorter latencies for downward as opposed to upward motion stimuli. For steady-state gain, there was a hint of U-D asymmetry that did not reach significance (*t*(16) = 1.582, *p* = 0.1332). Initial acceleration (*t*(16) = 3.384, *p* = 0.0038), steady-state gain (*t*(16) = 10.476, *p* < 0.0001), proportion smooth (*t*(16) = 4.085, *p* = 0.0009), and speed responsiveness (*t*(16) = 6.270, *p* < 0.0001) all showed a significant H-V asymmetry, resulting in smoother responses with more vigorous acceleration, more accurate gain, and greater speed sensitivity for motion stimuli along or near the horizonal meridian.

#### Octant analysis

**Figure 6:**
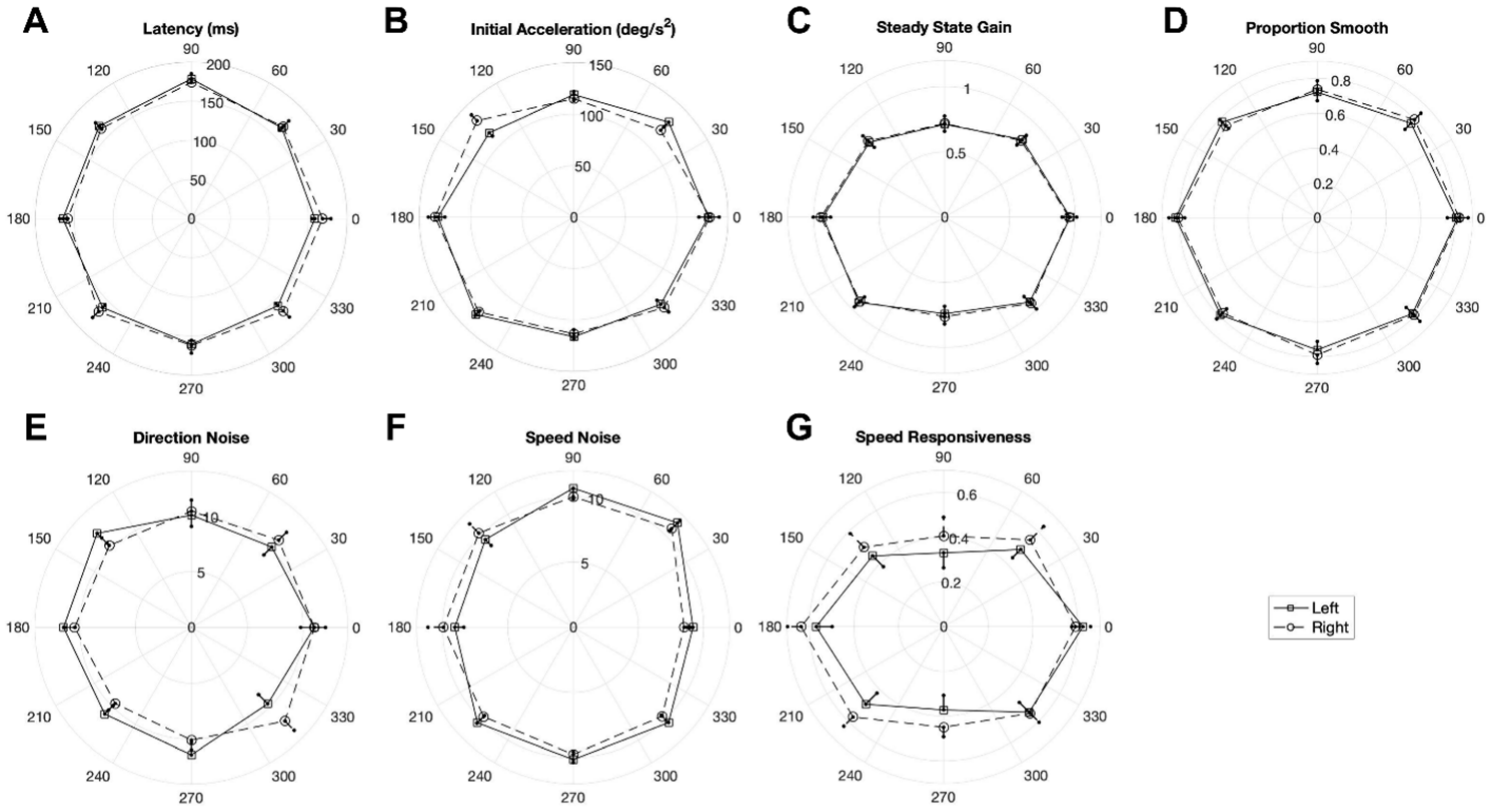
Polar plots of mean oculometric values for left and right eye viewing in world/head coordinates (based on the direction of ramp motion in the world) for **(A)** latency, **(B)** initial acceleration, **(C)** steady-state gain, **(D)** proportion smooth, **(E)** direction noise, **(F)** speed noise, and **(G)** speed responsiveness. 0 deg represents rightward ramp motion (following leftward steps). Error bars represent SEM.

Figure 6 illustrates polar plots of mean oculometric performance for left and right eye-viewing in world- or head-centric directional coordinates, subdividing the data into octants. The data indicate non-circular directional tuning for four of the seven pursuit-related oculometric measurements examined. Specifically, latency (χ^2^(14) = 31.599, *p* = 0.0024), steady-state gain (χ^2^(14) = 142.731, *p* < 0.0001), proportion smooth (χ^2^(14) = 24.577, *p* = 0.0390), and speed responsiveness (χ^2^(14) = 29.139, *p* = 0.0100) show significantly non-circular tuning. Initial acceleration was nearly significantly non-circular (χ^2^(14) = 23.129, *p* = 0.0582). While most of these oculometric measures show largely overlapping response curves for the left and right eyes, there is a significant N-T asymmetry (i.e., a systematic horizontal shift of the plot centers between the two eyes in Figure 6) for latency (*t*(16) = 2.256, *p* = 0.0384) and direction noise (*t*(16) = 2.281, *p* = 0.0366), expanding upon the less powerful quadrant analysis above.

**Figure 7:**
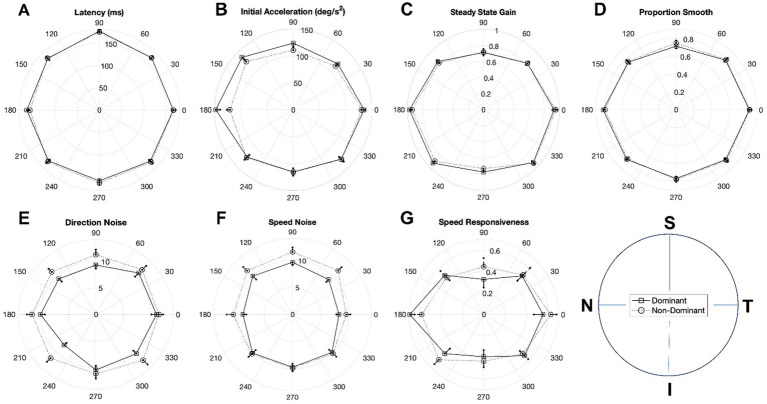
Polar plots of mean oculometric values for dominant and non-dominant viewing in retinal coordinates for **(A)** latency, **(B)** initial acceleration, **(C)** steady-state gain, **(D)** proportion smooth, **(E)** direction noise, **(F)** speed noise, and **(G)** speed responsiveness. Polar direction represents retinal locus of the stimulus motion during the analysis interval (see legend bottom right). Error bars represent SEM.

Figure 7 illustrates polar plots of mean oculometric performance for dominant- and non-dominant-eye viewing in retinal spatial coordinates, subdividing the data into octants. For 3 of the 7 oculometric measures, dominant- and non-dominant-eye viewing conditions generated significantly different plots within anisotropic differences: initial acceleration (χ^2^(7) = 18.345, *p* = 0.0105), direction noise (χ^2^(7) = 44.258, *p* < 0.0001), and speed noise (χ^2^(7) = 16.186, *p* = 0.0235). For acceleration, the dominant-eye viewing advantage appears predominantly for stimuli presented on the nasal retina (averaged across leftward and right motion), while for speed noise, the advantage appears predominantly in the superior retina (or downward motion). For direction noise, the advantage appears more widespread, albeit primarily in nasal and superior retina.

**Figure 8:**
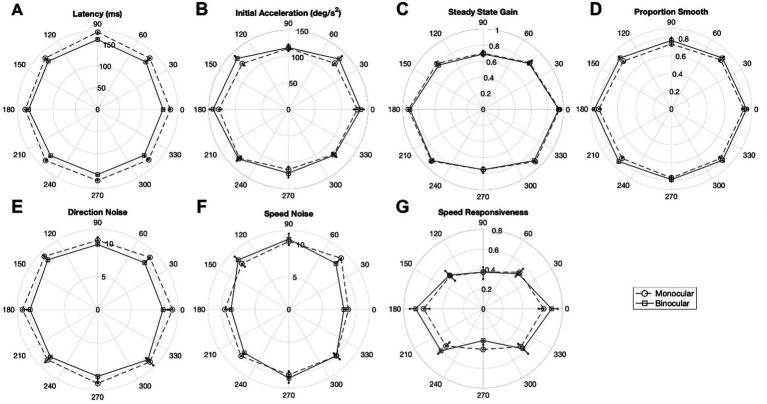
Polar plots of mean oculometric values for binocular and monocular viewing with respect to ramp direction in world/head coordinates for **(A)** latency, **(B)** initial acceleration, **(C)** steady-state gain, **(D)** proportion smooth, **(E)** direction noise, **(F)** speed noise, and **(G)** speed responsiveness. 0 deg represents rightward ramp motion (following leftward directed steps). Error bars represent SEM.

Figure 8 illustrates polar plots of mean oculometric performance for monocular and binocular viewing in world/head directional coordinates, subdividing the data into octants. First, we performed a circularity test of the binocular viewing curves and found that initial acceleration (χ^2^(7) = 14.8098, *p* = 0.03851), steady-state gain (χ^2^(7) = 62.3725, *p* < 0.0001), proposition smooth (χ^2^(7) = 14.9809, *p* = 0.0362), speed responsiveness (χ^2^(7) = 51.8991, *p* < 0.0001), deviate significantly from circularity with latency approaching significance (χ^2^(7) = 12.9522, *p* = 0.0733). A direct comparison between the monocular and binocular curves found that only 2 of the 7 above oculometric measures, generated significantly different plots: latency (χ^2^(7) = 64.9340, *p* < 0.0001) and proportion smooth (χ^2^(7) = 17.5391, *p* = 0.0142). Although the difference for direction noise did not reach significance with χ^2^ testing due in part to the relatively large across-subject variance, a simple coin-flip test would suggest it is highly unlikely that all 8 mean values were larger for monocular viewing by chance (*p* = 0.0039). Similarly, it is unlikely that 7 of 8 values of initial acceleration were lower for monocular viewing by chance (*p* = 0.0352). All these binocular enhancements appear to be largely isotropic scaling. Subdividing the data into octants lowered the statistical power such that some across-subject χ^2^ differences did not reach significance despite the significance overall within-subject differences observed when comparing average values across the whole data set in Table 2.

## Discussion

We have investigated the effects of three viewing conditions on human ocular tracking behavior (left-eye only, right-eye only, and both eyes) to determine the influence of binocularity and line-of-sight eye dominance on visually-driven oculomotor performance. Our results show that binocular viewing generates systematically superior tracking performance compared to monocular viewing (Table 2 and Figure 2). Not surprisingly, combining information across both eyes improves a wide array of features of visuomotor processing. Smooth-pursuit initiation is faster (lower latency) and more vigorous (higher initial open-loop acceleration), thereby reducing the need for saccadic compensation (lower catch-up saccade rate and amplitude, and higher steady-state proportion smooth). The pupillary light response is also faster and more robust (reduced time constants and smaller average diameter) because two eyes collect nominally twice as much light as one. We also found two additional differences without clear benefit: increased direction asymmetry and altered saccade generation (decrease slope of the main sequence), the latter of which may just be an artifact of somewhat unstable vergence during monocular blindfolding. On the other hand, line-of-sight eye dominance is associated with the selective improvement of visual motion processing with the dominant eye providing higher open-loop signal-to-noise via increased initial acceleration, decreased direction noise, and to a lesser extent decreased speed noise (Table 3 and Figures 3, 7). The 15 other features of visuomotor processing that we tested remain largely the same for dominant- and non-dominant-eye viewing. A comparison of binocular performance with dominant-eye monocular performance allowed us to isolate the effects of binocularity *per se* (separate from those due to the simple fact that binocular viewing always includes access to information from the dominant eye, while monocular cyclopean performance only gets half its information from the dominant eye). From this comparison (Table 4 and Figure 4), we conclude that binocularity specifically decreases response latency, reduces saccadic responses, augments pupillary light responses, and appears to systematically increase the horizontal bias of direction accuracy as well (higher direction asymmetry).

In addition to the above overall findings, we performed a spatial/directional analysis on a subset of seven pursuit-related oculometrics (latency, acceleration, gain, proportion smooth, direction noise, speed noise, and speed responsiveness) during monocular viewing to determine to what extend the responses were isotropic across retinal locus and/or visual direction. First, we performed an initial analysis of the left and right eye viewing data segregated into quadrants and found a number of anisotropies (Table 5). Latency was found to have a N-T asymmetry, with nasal retina generating faster pursuit responses, and a U-D asymmetry, with superior retina showing more delayed responses to upward motion in lower visual field. Initial acceleration, steady-state gain, proportion smooth, and speed responsiveness showed clear H-V asymmetries with horizontal responses being systematically more vigorous, more accurate and sensitive to speed variation, and smoother than vertical responses. Second, we performed a higher resolution polar analysis of the data segregated into octants (Figure 6) and found deviations from circular symmetry for four metrics (latency, gain, proportion smooth, and speed responsiveness) with initial acceleration showing a hint of non-circularity. Note that, while averaged over both eyes, the precision of direction and speed processing appears largely circularly tuned (Figure 8; Krukowski and Stone, 2005), direction precision (along with latency) nonetheless exhibits a N-T bias, evident in a systematic horizontal displacement of the circular tuning between the left and right eye plots (Figure 6), with nasal retina generating better performance. Thirdly, dominant-eye viewing appears to generate anisotropic enhancement of motion processing with acceleration enhanced predominantly for nasal retina, with direction precision enhanced predominantly for nasal and superior retina, and with speed precision showing improvement predominantly for superior retina (Figure 7) such that the overall effect across all directions on speed precision does not reach significance (Table 3). Lastly, four metrics (latency, acceleration, direction noise, and proportion smooth) show largely isotropic differences between the polar plots for binocular and monocular viewing based on χ^2^ and coin-flip statistics (Figure 8), consistent with the overall binocular superiority discussed above (Table 2). Our findings are summarized in Table 6.

**Table 6:** Summary of the main findings for the effects of line-of-sight (LOS) eye dominance and binocularity.

		LOS-dominance	Binocularity
Early Perifoveal Vision	Latency (ms)	↔	**↓**
	Acceleration (deg/s^2^)	**↑**mostly Nasal/Superior	↔
Late Foveal Vision	Gain	↔	↔
Visual Motion Precision	Direction Noise (◦)	**↓**mostly Nasal/Superior	↔
	Speed Noise (%)	**↓** Superior only	↔
Visual Motion Accuracy	Direction Anisotropy	↔	↔
	Direction Asymmetry	↔	**↑** more H bias
	Speed Responsiveness	↔	↔
Subcortical Vision	Saccade Rate (Hz)	↔	**↓**
	Saccade Amplitude (deg)	↔	**↓**
	Saccade Dispersion (◦)	↔	↔
	Proportion Smooth	↔	**↑**
Pupillary Response	Contraction τ (ms)	↔	**↓**
	Dilation τ (ms)	↔	**↓**
	Pupil Diameter (mm)	↔	**↓**
Brainstem Saccade Generator	Main Sequence Slope (Hz)	↔	**↓?**
	Main Sequence Intercept (deg/s)	↔	↔
Localization	Fixation Error (deg)	↔	↔

Note that these two viewing conditions impact our oculometrics differently with the former affecting early retinal motion processing with clear spatial/directional tuning in retinal coordinates and the latter affecting later visual processing largely isotropically (except for the altered horizontal vs. vertical bias captured by the Direction Asymmetry metric). Note also that other phenomena (e.g., the oblique effect captured by the Directional Anisotropy metric) appear unaffected by eye dominance or the number of eyes viewing the stimulus. The larger bolded arrows indicate larger effects.

### Potential relationship to prior structural and physiological findings

In a cohort of 112 healthy participants, Sabouri et al. (2016) measured and reported the retinal nerve fiber layer thickness for each parafoveal quadrant. The mean (±SD) thickness of the parafoveal nasal sector (N3) was 319.7 (±15.0) μm and of the temporal sector (T3) was 306.0 (±15.6) μm. Thus, in their large cohort of healthy subjects, nasal parafoveal retina is 13.7 μm thicker than its temporal counterpart (which would result in *p* < 0.01, t-test) (Sabouri et al., 2016). There was no obvious thickness difference between the superior and inferior quadrants, or the horizontal and vertical quadrants. It is tempting to associate their observed structural N-T asymmetry with our observed behavioral N-T asymmetry whereby pursuit initiation is enhanced for motion stimuli on nasal versus temporal parafoveal retina. This intriguing potential link invites further examination of the correlation (if any) between thickness and performance (whether due to natural or pathological variation) on a quadrant-by-quadrant basis. There also appears to be significant N-T asymmetry in multifocal electroretinogram responses in healthy adult subjects with the amplitude ratio of the P2 to P1 waves larger in the temporal field (Viswanathan and Demirel, 2002) and sensitive to retinal pathology (Moroto et al., 2023). Thus, the confluence of the behavioral, physiological, and anatomical differences between nasal and temporal retina supports the notion that the neural processing of motion information in these two retinal loci may be different, although a systematic within-subject correlation study is needed to determine if these three phenomena are reliably related.

### Previous relevant findings on oculomotor effects of viewing condition

While we know of no prior systematic evaluation of the effects of viewing conditions specifically on the ocular tracking of step-ramp stimuli, as part of a study on the effects of macular degeneration on pursuit, Shanidze et al. (2017) examined the pursuit responses to step-ramps in a small (*N* = 4) cohort of healthy (albeit older) control subjects, with their highest speed tested (15 deg/s) very similar to our slowest stimulus (16 deg/s). Their ANOVA found no significant difference in pursuit gain between three viewing conditions (their Figure 2), including between monocular vs. binocular viewing, consistent with our findings in Table 2, and between line-of-sight dominant vs. non-dominant viewing, consistent with our findings in Table 3. They did not report other oculometric measures that we can compare with ours.

Other previous studies have found effects of eye dominance on visual and visuomotor performance, examining manual and ocular tasks under conditions quite different from our study (e.g., Kolesnikova et al., 2010; Bargary et al., 2017; Verghese et al., 2021). In an extensive oculomotor study with over thousand participants, Bargary et al. (2017) reported only a weak relationship between an ipsi-contra hemispheric asymmetry of the latency of voluntary saccades and line-of-sight eye dominance. An earlier study had reported a link between eye dominance and saccadic latency asymmetry (Vergilino-Perez et al., 2012), but the same group later clarified that this effect appears modulated by the “strength” of a non-binary eye dominance (Chaumillon et al., 2017), thus potentially reconciling the findings of these two groups. Bargary et al. (2017) made no mention of any other eye-dominance effects on pursuit or saccades, despite an extensive examination of nearly two dozen oculometric parameters, consistent with our general finding that most oculometric parameters are insensitive to line-of-sight eye dominance. However, although they did measure pursuit initial acceleration, they did not report any effect of eye dominance. The presumed absence of any effect of eye dominance on initial pursuit acceleration in their study conflicts with ours and could be due to the fact they used a periodic, entirely predictable, triangle-wave tracking stimulus so their observed tracking responses were likely dominated by a top-down predictive input as opposed to the responses in our task that are driven largely by bottom-up visual motion processing, due to the high spatio-temporal uncertainty of our stimulus paradigm (Krukowski and Stone, 2005). Bargary et al. (2017) did not examine direction or speed noise.

Finally, a study of micro-saccades during fixation under binocular versus monocular viewing condition found little change in the main sequence (Essig et al., 2020). However, they did observe small differences that they attributed to small loss of binocular eye alignment just as we suggest above for our small catch-up saccades. Lastly, a study of fixational stability during foveal viewing found no difference between dominant, non-dominant, and binocular viewing (Hirasawa et al., 2018), consistent with our results.

### Previous findings on directional tuning

Previous studies of human pursuit response have examined specific aspects of directional tuning of ocular pursuit. In an early study of the pursuit response to two-target 30 deg/s ramp motion (similar to our step-ramp stimuli except slightly faster than our fastest stimulus and with greatly reduced uncertainty) in the four cardinal directions centered on the primary quadrants under monocular viewing (half with left and right eye viewing collected, half with dominant-eye only), Tychsen and Lisberger (1986a) examined a small cohort of healthy adults (*N* = 6) and found a large centripetal enhancement in initial acceleration for horizontal tracking that peaked around 3 deg eccentric (close to that used for all our stimuli), but reported no N-T asymmetry, consistent with our findings in Table 5 and Figure 6, although they did subsequently report a severe N-T asymmetry in humans with naturally occurring early-onset strabismus (Tychsen and Lisberger, 1986b), which is also seen in monkeys with induced strabismus (Kiorpes et al., 1996; Joshi et al., 2017). Tychsen and Lisberger (1986a) also reported no U-D asymmetry during vertical tracking (although there were clear indications of a small downward bias in their data, e.g., their Figure 2B), but instead found a large enhancement for motion in either direction in the lower visual field (superior retina). Although they rigorously demonstrated that the initial 100-ms of acceleration indeed captures the *bone fide* open-loop visual drive for pursuit (their Figure 10), unfortunately, they did not assess their latency data for any spatial/directional tuning, nor did they perform statistical tests to confirm or rule out subtle directional asymmetries. In another study of pursuit responses to step-ramp stimuli in 8 possible directions (cardinals and primary obliques) during monocular dominant-eye viewing, Rottach et al. (1996) reported idiosyncratic asymmetries that differed across their small subject pool (*N* = 5) and across ramp speeds, but no consistent L-R, U-D, or H-V asymmetries in either latency or initial acceleration. These negative findings however could be due contamination of their latency and acceleration measures by their reported initial transient artifactual pursuit response to apparent step “motion” in the opposite direction of the ramp caused by a sluggish mirror galvanometer projection system. This artifact was expressly avoided in Tychsen and Lisberger (1986a) with their two-target paradigm and here with the high frame rate of our modern gaming display.

In a more recent systematic study of direction tuning on a larger sample of healthy adults (*N* = 20), Ke et al. (2013) examined pursuit responses to step-ramps during binocular viewing, under conditions similar in principle to ours (their Experiment 1) although they only tested 8 directions corresponding to the centers of the primary octants while we tested 90 directions across the full range of possible directions (increasing spatial/directional uncertainty) and then clustered them into octants. Using repeated-measures ANOVA across their 8 directions, they found significant directional effects on latency, gain, and smoothness (their Table 1), consistent with our χ^2^ analysis across octants in Figure 8, although for us latency did not quite reach significance. Their directional effect on initial acceleration did not quite reach significance (ours did). Their post-hoc t-testing found that their observed U-D asymmetry (downward preference) for latency was not quite significant (ours was), for steady-state gain was significant at higher speeds (their Table 2) (ours approached significance), and for smoothness was significant (ours was not). The last difference was likely methodological; their smoothness measure (saccade number) included any initial larger saccade during tracking onset, while our smoothness measure is restricted to true steady-state smaller catch-up saccades. Their observed U-D asymmetry of smoothness may be an indirect consequence of an U-D asymmetry in latency putting gaze further behind the target at trial onset, thus increasing the likelihood of early saccades (their finding), which then washes out in the steady-state (our finding). They also found a significant H-V directional asymmetry for gain (their Table 2) as did we, but surprisingly not for initial acceleration or smoothness as we found.

In their Experiment 1 (Ke et al., 2013) and ours, because we both used only central fixation and centripetal ramps, one cannot distinguish between directional tuning and effects of retinal locus. However, their Experiment 2 specifically controlled for this by comparing step-ramps presented in the upper versus lower visual field. They found that the latency asymmetry we both observed appears due to retinal (visual field) locus with the inferior retina generating faster responses. They also found a significant true directional U-D asymmetry in steady-state gain, independent of retinal locus. Their evidence for a true directional U-D asymmetry in initial acceleration was however weaker, as it was only true in the upper visual field (on the inferior retina). Despite the fact that there was no directional uncertainty and half of the trials were at a much larger eccentricity in their control experiment, it is fair to say they showed that the vertical asymmetry for steady-state gain is indeed likely due primarily to an U-D directional anisotropy *per se*, although for us this effect did not quite reach significance. From their findings and those of Tychsen and Lisberger (1986a), latency and initial acceleration however appear highly influenced by retinal (visual field) locus. Perhaps the pitting of opposing directional and retinal factors (enhancement for downward motion and diminishment for superior retina during steady-state tracking, and vice versa) in our centripetal-only test paradigm might explain why the U-D asymmetry in steady-state gain in our study did not reach significance.

Our direction tuning findings under binocular viewing conditions confirm the key findings by Ke et al. (2013) and complement them by finding additional effects but, more importantly, we also fundamentally extend them by demonstrating the existence of anisotropies during monocular viewing and showing that these anisotropies are influenced by eye dominance. In particular, the N-T asymmetries that we describe for the first time are inherently in retinal coordinates (explicitly in Table 5 and implicitly in Figure 6) and thus are directly due at least in part to the spatial tuning of retinal processing. The selective enhancement of initial acceleration and reduction in direction noise for nasal retina and potentially the reduction in speed noise for superior retina with dominant eye viewing (Figure 7) are also due to spatial tuning in retinal processing.

### Previous relevant findings on pupillary effects of viewing condition

There are many prior systematic and thorough studies of the effects of viewing condition and other factors (monocular vs. binocular viewing, luminance, field size, age, adaptation state, cognitive workload, etc., for a review, see Watson and Yellott, 2012) on pupillary light responses. Using the Watson-Yellott pupil calculator, which captures many of these factors, the anticipated steady-state pupil diameter under our conditions (median age: 35 years, effective field diameter: 50 deg, mean luminance: 49 cd/m^2^) is 4.3 and 3.2 mm for monocular and binocular viewing, respectively. Our empirical median values are somewhat smaller (3.3 and 2.9 mm, respectively) but this is likely because we measured mean pupil size using a luminance square-wave duty-cycle of ~0.3 Hz, which is too fast to allow the pupil to reach full steady-state. Given that dilation is more sluggish than contraction and that our background lighting outside of the display was not completely dark, one would indeed anticipate our median values to be somewhat more constricted than the predicted steady-state diameter.

Previous studies have proposed that the effect of binocular summation can be explained by a forward shunting inhibition model (ten Doesschate and Alpern, 1967; Varjú, 1967a,b; Clarke et al., 2003), which predicts that the binocular steady-state constriction response is sub-additive and related to the monocular steady-state step response by Equation 1 with a shunting strength (k) of ~0.2 (in units of mm^−1^).

(1) $$B=\frac{2\;M\;}{\left(1+kM\right)}$$

If one assumes a dark pupil diameter of 6.9 mm (per both Watson and Yellott, 2012; Clarke et al., 2003), our observed median monocular (M) and binocular (B) constriction responses of 3.6 and 4.0 mm, respectively, correspond to a shunting strength of 0.22. Furthermore, if one assumes that the pupillary light response can be broadly characterized using first-order dynamics as we did when we measured our time constants as opposed to the more complete third-order dynamics used by others (Stark and Sherman, 1957; ten Doesschate and Alpern, 1967; Varjú, 1967a,b; Clarke et al., 2003), the shunting model predicts that the binocular time constant (*τ*_b_) will be similarly shortened relative the monocular time constant (τ_m_) per Equation 2.

(2) $$\tau_{b}\sim \frac{\tau_{m}\;}{\left(1+kM\right)}$$

From our data, the above simple relationship between our monocular and binocular time constants predicts shunting strengths somewhat lower than expected (0.035 and 0.079, for constriction and dilation, respectively). To ensure that our simplification of the model dynamics did not distort this result, we also examined the predicted relationship between τ_b_ and τ_m_ for the third-order model used by Clarke et al. (2003) which assumes the cascading of a first-order low-pass filter and a second-order damped oscillator. Interestingly, the measured median value of the first-order time constant for our binocular response was 156 ms and the best-fitting time constant of the first-order filter of their model was 150 ms, showing remarkable agreement. We then derived the expected third-order monocular response by running Clark and colleagues’ binocular model through the inverse of the shunting model to yield the equation in Laplace space of the denominator of the monocular transfer function. The inverse of the real root of Equation 3 is τ_m_.

(3) $$s^{3}\;\frac{2\tau_{b}\;}{{\left(2\pi f\right)}^{2}}+s^{2}\left(\frac{4D\tau_{b}}{2\pi f}+\frac{2\;}{{\left(2\pi f\right)}^{2}}\right)\;+s\left(2\;\tau_{b}+\frac{4\;D\;}{2\pi f}\right)\;+\left(2-kB\right)=0$$

Using our observed median values of τ_b_ (156 and 588 ms for constriction and dilation, respectively) and B together with their best-fitting damping coefficient (*D* = 0.7) and resonant frequency (*f* = 1.3 Hz), we recursively solved Equation 3 (using Wolfram Alpha™) and found that the best-fitting k for constriction was 0.029 and for dilation was 0.098, which are comparable to their respective simplified first-order values derived above. It should be mentioned that k would be higher if the shunting inhibition were allowed to have sluggish dynamics (i.e., k had its own low-pass dynamics as opposed to being a constant). That would explain both the fact that k during the transient responses is lower than the expected steady-state value of ~0.2 and that the value is of k is higher for dilation than for constriction, as the former is more sluggish thus allowing more time for shunting to kick in. In summary, although our single-condition on–off step-response tests of the pupillary light reflex under monocular and binocular viewing is insufficient to provide compelling evidence for any specific model of binocular summation, our findings are nonetheless largely consistent with the forward inhibition shunting model, and perhaps other models that also predict sub-additive summation across the two eyes (in our case, an 11% increase) and a decrease in the system time constant (in our case, 11% for constriction and 22% for dilation). A systematic examination of the time course of the pupillary response across luminance and other conditions will be needed to determine if the shunting model can reliably predict the relationship between the monocular and binocular time constants with a well-behaved gain. Lastly, previous studies have found that the direct and consensual pupillary responses, driven by the stimulation of the measured and non-measured eye, respectively, are the same (see Clarke et al., 2003). This suggests the absence of asymmetric inputs from the two eyes, thus precluding any eye-dominance effect, consistent with our findings.

### Caveats

There are limitations to this study, one which is the subjective nature of the Miles eye-dominance test and thus the potential for erroneous categorization (Miles, 1930). If such errors occurred with our cohort, it would only serve to decrease the significance of any observed effects so our positive results cannot be due to categorization errors. In addition, there are many different types or measures of eye dominance, some related to acuity and sensory rivalry as well as sighting (for a review see, Mapp et al., 2003) and eye dominance may not be a binary phenomenon (Vergilino-Perez et al., 2012; Chaumillon et al., 2017). In this study, we only examined line-of-sight dominance which we treated as a binary variable, i.e., we did not try to determine the strength of this dominance, so our conclusions are limited to the effects of binary line-of-sight dominance.

Second, extended (~2 h) monocular deprivation can temporarily alter sensory dominance (Ooi and He, 2020). In our study, although we did monocularly deprive (blindfold) participants during monocular testing, the duration of blindfolding was very brief (<10 min) and we randomized the order of the testing, and therefore blindfolding, of the left and right eyes, so it is highly unlikely that our procedures produced any meaningful plasticity and our design counterbalanced for any such effects. Lastly, when we empirically compared the performance when the dominant eye was blindfolded first versus second, we did not find a significant difference (*t*(14) < 0.47, *p* > 0.32). Thus, it is highly unlikely any plasticity occurred during blindfolding that could have influenced our findings.

Third, we also did not rigorously control for circadian rhythm, which is a confounding factor known to affect human ocular tracking responses (Stone et al., 2019). However, all testing was performed on well-rested participants at similar times in the morning (between 7:00 am and 10:00 am) therefore any such confound was greatly minimized.

Fourth, our design did not allow us to distinguish between direction and retinal (visual field) locus as the cause of tuning effects because our stimulus motion was always centripetal. However, Ke et al. (2013) directly examined this issue using both centripetal and centrifugal vertical stimuli by varying the location of the stimuli in the visual field. They found that the anisotropies in latency were associated with retinal locus (visual field) while all the other U-D asymmetries they observed appeared directional (their Table 3) although Tychsen and Lisberger (1986a) found that initial acceleration was primarily influenced by retinal locus (their Figure 2B). Although it is reasonable to extrapolate these studies to our findings with caution (see above), because our stimuli were restricted to centripetal motion, we cannot be absolutely sure our observed S-I retinal asymmetry in latency was not actually an U-D directional asymmetry. However, that said, by combination left and right eye viewing (and counterbalancing direction), we could indeed establish a *bone fide* N-T retinal locus effect without a directional confound.

Fifth, we measured eye movements monocularly (through the viewing eye for monocular data and through the left eye for binocular data) so we did not measure the extent to which the motor response of the two eyes might have been different. That said, regardless of viewing condition, the conjugate versional movements of the two eyes are tightly yoked by connections between the abducens and oculomotor nuclei that cross the midline through the medial longitudinal fasciculus (Bhidayasiri et al., 2000; Frohman et al., 2008; Leigh and Zee, 2015) so it should not matter which eye is physically tracked as long as tracking remains within ~15 deg of central gaze on a fronto-parallel plane (as was the case in our study). Furthermore, Ke et al. (2013) found no significant difference between data collected from tracking either the left or the right eye, confirming this principle empirically under similar conditions. That said, binocular viewing does have a small effect on the main sequence, likely by keeping vergence better locked, while monocular viewing may impair ocular alignment by eliminating visual feedback from the blindfolded eye. Resolution of this intriguing latter detail awaits a binocular tracking study.

Sixth, our limited measurement of pupillary responses is not intended to fully characterize the pupillary light reflex with its multiple pathways and complex input–output characteristics (Varjú, 1967a,b; ten Doesschate and Alpern, 1967; Clarke et al., 2003; Gamlin et al., 2007; Watson and Yellott, 2012; Belliveau et al., 2024; Mure, 2021; Barrionuevo et al., 2023). The collection of the pupillary response to a couple of cycles of square-wave modulation of white light at the end of an extended exposure to a constant luminance background during our eye-tracker calibration procedure was merely designed to generate reproducible pupillary responses that can be used to detect gross differences across test conditions or cohorts (e.g., Tyson et al., 2021).

Lastly, although our participant group was larger and more diverse than most previous step-ramp ocular tracking studies, it remains a relatively limited compared to the general population, so we may well have missed subtler but important effects and extrapolation to the general population must be made with caution. That said, our binocular-viewing data set (Table 2) is very similar to that from a prior normative data set from a completely different healthy cohort of 41 participants who were not as rigorously screened, not necessarily well-rested, but were tested using the identical stimulus paradigm albeit with a 60-Hz monitor (Liston et al., 2017). For the 8 metrics that were computed similarly (correcting the older latency data to be respect to motion onset instead of step onset by subtracting a single frame duration), the median difference was less than 8% and linear regression of the two sets of median values yielded a slope of 1.05 and *r^2^* of 0.998, with a slight tendency for better performance in the current cohort. With the small-cohort caveat in mind, tempered by the similarity across two independent cohorts, our data can nonetheless serve as a baseline control group for future across-subject studies, as long as the test group shares well-matched screening and demographics (Berneshawi et al., 2024).

## Conclusion

Our findings demonstrate that many features of human eye movements are influenced by viewing conditions and, in particular, by both line-of-sight eye dominance and binocularity. Binocularity makes pursuit and pupillary responses faster and reduces the need for saccadic compensation, as one would predict from signal detection theory when two independent sources of information are available. Line-of-sight eye dominance specifically improves the open-loop direction response amplitude and precision showing that visual motion processing through the dominant eye is more robust with higher signal-to-noise. We also found that these eye-dominance enhancements are largely anisotropic in retinal coordinates. We conclude that certain functional anisotropies in visual motion processing (e.g., the N-T asymmetries in latency and initial acceleration) are retinal in origin and may be related to known normative structural anisotropies in retinal thickness (Sabouri et al., 2016). Conversely, the increase with binocular viewing in the H-V bias of the direction estimate (direction asymmetry) driving pursuit initiation suggests interesting non-linear cortical processing that presumably has an evolutionary value when living in earth’s gravitational environment. Finally, while our binocular normative dataset allows for across-subject comparisons with mildly impaired performance due to physiological or environmental factors in general, our monocular dataset, segregated by eye dominance, will enable more sensitive detection of mildly altered visuomotor performance driven by a pathological retina by controlling for this important factor.

## Data Availability

The datasets presented in this article are not readily available however anonymized data will be provided by request to the extent authorized by NASA and its human research institutional review board. Requests to access the datasets should be directed to the corresponding author at Leland.S.Stone@nasa.gov.
